# Causes of community deaths by verbal autopsy among persons with HIV in 33 districts in Zambia, 2020–2023

**DOI:** 10.1371/journal.pone.0338453

**Published:** 2025-12-17

**Authors:** Priscilla Kapombe, Choolwe Jacobs, Mark W. Tenforde, Kashala Kamalonga, Diane Morof, Terrence Lo, Mweene Cheelo, Lloyd Mulenga, Sombo Fwoloshi, Cordilia M. Himwaze, Patrick Musonda, Mpundu Makasa, Jonas Z. Hines

**Affiliations:** 1 Division of Global HIV & TB, U.S. Centers for Disease Control and Prevention, Lusaka, Zambia; 2 University of Zambia, Lusaka, Zambia; 3 Ministry of Health, Lusaka, Zambia; 4 Ministry of Home Affairs, Lusaka, Zambia; Newlands Clinic, ZIMBABWE

## Abstract

Zambia has achieved improvements in life expectancy among persons living with HIV (PLHIV) because of high antiretroviral therapy (ART) coverage, which should improve survival due to reductions in AIDS-defining conditions. However, recent estimates of the most common causes of death are not widely available. We utilized mortality surveillance data to report on common causes of death among persons with HIV who died in community settings in Zambia. The Zambian Ministry of Health conducted sentinel mortality surveillance of community deaths in 45 hospitals in 33 of 116 districts from January 2020 through December 2023. Verbal autopsies (VA) were conducted through interviews with relatives or close associates of deceased persons using the 2016 World Health Organization tool. HIV status was reported. A probable cause of death was assigned by a validated computer algorithm (InterVA5). We describe the top assigned causes of death stratified by HIV status. Verbal autopsies were conducted for 67,079 community deaths, of which 11,475 (17.1%) were persons with HIV. The mean age at death was 45 years among persons with HIV and 48 years for persons without HIV (T-test p < 0.001). The most common probable causes of death identified by VA among persons with HIV were HIV/AIDS-related causes (50.4%), cardiac disease (13.1%), pulmonary tuberculosis (7.5%), and digestive neoplasms (3.9%)**.** Leading probable causes in decedents without HIV were cardiac disease (24.9%), stroke (8.5%), and acute respiratory infection including pneumonia (7.6%). Overall, the percentage of deaths attributed to HIV/AIDS-related causes decreased from 11.2% to 7.5% (trend test p < 0.001) and the percentage of cardiac deaths increased from 18.7% to 25.4% (p < 0.001) from 2020 to 2023. These findings support interventions to strengthen the integrated management of noncommunicable diseases in community settings across Zambia. Among persons with HIV, a high percentage of deaths attributed to HIV/AIDS highlights the need to maintain high ART coverage and retention. Strategies, such as increased use of minimally invasive tissue sampling, may improve characterization of the high proportion of deaths attributed to non-specific HIV/AIDS-related causes through VA surveillance.

## Introduction

The rollout of universal antiretroviral therapy (ART) in populations with generalized HIV epidemics has greatly improved the survival of people living with HIV (PLHIV) in sub-Saharan Africa [1], including in Zambia where there are an estimated 1.4 million PLHIV [2]. This may shift causes of death (CoDs) from AIDS-defining conditions to other causes, including noncommunicable diseases, that increase in prevalence with age and may be accelerated in PLHIV [3]. AIDS-related conditions were the leading CoDs among adults in Zambia according to the 2010–2012 Zambia Sample Vital Registration with Verbal Autopsy (SAVVY) survey (accounting for 40.7% of all deaths), although more recent data are needed to understand changes over time [4]. Understanding CoD over recent time periods can be used to tailor appropriate clinical interventions and public health programs for PLHIV.

In Zambia, approximately 60% of the population live in rural settings where gaps exist in registering and ascertaining CoDs since health facilities may be difficult to access [4,5]. In urban settings, a large proportion of deaths also occur outside of health facilities or in the community, so would not typically be captured by existing vital statistics programs that source data from facilities [5]. Results from past surveys estimate that more than 40% of all deaths in Zambia occur outside of health facilities [6]. To address the gap in understanding CoD for deaths occurring within the community, since 2020 the Zambia Ministry of Health – with support from the U.S. Centers for Disease Control and Prevention (CDC) – has implemented sentinel mortality surveillance focused on community deaths within high HIV burden districts. Community deaths in Zambia, referred to as “Brought-in-Dead” (BID), are defined notionally as deaths occurring in the home or other community settings brought to a hospital mortuary as well as deaths occurring within 24 hours of admission to a health facility, on the assumption that extensive examination to determine CoD has not been achieved.

There are several methods for determining CoD, which are typically performed for deaths that occur within health care facilities, including the gold standard pathologist-determined CoD using complete pathologic autopsy, medical certification of cause of death (MCCD) by the attending clinician at the time of death, or physician panel assignment of CoD through review of medical records. Other methods, including minimally invasive tissue sampling, have also been used to collect additional granular data on CoD among persons with HIV and other populations in resource-limited settings [7–9]. Verbal autopsy (VA) is a process in which a respondent who knew the deceased, typically a family member or caretaker, completes a structured interview by a trained interviewer using a standardized questionnaire assessing signs, symptoms, and relevant events during the period leading to death [10]. From this information, a probable CoD is assigned either through a physician’s review or by an automated algorithm. In Zambia, VA has been used to monitor mortality trends for deaths that occur within the community in 33 districts within 7 out of 10 provinces, including those with high HIV prevalence, and has been established as an alternative source of mortality data for population lacking other reliable sources of mortality information [10,11].

We ascertained probable CoD and identified conditions associated with HIV-related deaths among persons with HIV in Zambia who died within the community or were brought in dead from 2020–2023 to inform clinical management and public health programs.

## Methods

### Study design

We performed a cross-sectional analysis of routine mortality surveillance data for deceased community members whose death underwent VA between 01/01/2020 and 31/12/2023. The Mortality Surveillance program (verbal autopsy data collection process) began in January 2020 and is currently ongoing. Data was accessed on 29/01/2024 for this analysis purposes.

### Study setting

This covered the following 7 provinces (33 districts) of Zambia that conducted mortality surveillance in 45 hospitals within these districts, during this time period: Copperbelt (Chingola, Kalulushi, Kitwe, Luanshya, Mufulira, Ndola); Eastern (Chipata, Katete, Petauke); Lusaka (Chongwe, Lusaka, Kafue); North-Western (Solwezi); Southern (Mazabuka, Chikankata, Chikuni, Choma, Kalomo, Kazungula, Livingstone, Maamba, Macha, Monze, Njase, Pemba, Siavonga, Sinazongwe); Western (Kaoma, Senanga, Sesheke, Mongu); and Central (Kabwe, Kapiri-Mposhi). These districts account for approximately 46% of the Zambian population. Cumulatively, the hospitals that implemented mortality surveillance are as follows:

Copperbelt: Ndola Teaching Hospital (TH), Kamuchanga District Hospital (DH), Thomson DH, Ronald Ross General Hospital (GH), Nchanga North GH, Kitwe TH, Arthur Davidson CH, Kalulushi GH, and Roan Antelope GH.

Eastern: Chipata CH, St Francis MH, and Petauke DH.

Lusaka: University Teaching Hospital, Levy Mwanawasa UTH, Kafue GH, Chongwe DH, Kanyama GH, Chelstone zonal clinic, Chilenje level one clinic, Matero level one hospital, Chawama level one, Chongwe DH, Kafue GH, and Nangongwe clinic.

North-Western: Solwezi GH.

Southern: Mazabuka GH, Choma GH, Kalomo DH, Livingstone TH, Monze MH, Namwala DH, Chikankata DH, Macha Mission, Siavonga GH, Kazungula DH, Chikuni Mission, Njase Clinic, Pemba main clinic, and Maamba DH.

Western: Senanga GH, Kaoma GH, Lewanika GH, and Yeta DH

Central: Kapiri Urban Clinic and Kabwe General Hospital

### Study population and sampling

All decedents across all age groups were included in this analysis.

### Data collection

Mortality surveillance officers conducted VAs with relatives, caretakers, or close associates of deceased persons who were brought to a health facility mortuary. Using an electronic version of the 2016 World Health Organization standardized tool in ODK, VA was performed for persons who died in the community or who died within 24 hours of arrival at the hospital or other health facility (persons dying >24 hours after hospital admission had a MCCD form completed by the attending provider). Data were first collected at facility level then synced to a central server (Kobo Collect Server).

### Data management and analysis

The VA questionnaire instrument was designed for all age groups, and specific questionnaires were available for three age categories: under four weeks (categorized as “Neonates”); between 4 weeks and 11 years (categorized as “Children”), and 12 years and above (categorized as “Adults”).

Based on responses to the VA questionnaire, a probable CoD was assigned on a data management platform called Verbal Autopsy Explorer (VAE) using InterVA5, a validated computer algorithm [11] in OpenVA. The InterVA mapped causes of death using International Classification of Diseases, Tenth Revision (ICD-10) codes [11]. All information in the VA was reported by the respondent and no laboratory testing was performed. However, if medical records were available, the mortality surveillance officer attempted to capture this information (e.g., HIV testing results or clinical diagnosis) during VA. Data were aggregated in dashboards for predetermined variables of interest and Excel files could be downloaded by authorized personnel with all questionnaire variables and the narrative for further detailed analysis.

We defined HIV-positive status for a decedent as: 1) respondent reported positive HIV status or a health professional diagnosis of AIDS for the deceased person; 2) respondent reported deceased person being on antiretroviral therapy (ART) at the time of death; 3) mention of “HIV”, “AIDS”, or “RVD” (retroviral disease) as a cause of death by a provider to the respondent; and/or 4) InterVA5 assigned probable cause of death of “HIV/AIDS related causes.” HIV-negative and unknown HIV status were defined by the response to the question about HIV status [11]. Acute cardiac disease (code 04.01) and other and unspecified cardiac disease (code 04.99) causes of death were combined into one classification, “cardiac disease,” to understand the broader impact of cardiac conditions.

We analyzed patient demographics, medical history, and symptoms proximal to death descriptively using counts and percentages or median and interquartile range, as indicated, stratified by HIV status. Differences in distributions between decedents with and without HIV were compared using Welch’s T-test for age and Pearson’s chi-square test or Fisher’s Exact test for categorical variables. We evaluated changes over time for commonly assigned probable CoD. To assess whether there were trends in the prevalence of the most common CoDs over time, overall and stratified by HIV status, we conducted Cochran-Armitage trend tests. A p-value <0.05 was considered statistically significant. Analysis was performed using SAS Version 9.4, R Version 4.3.2 and STATA Version 18.

The study protocol was approved by the ERES Converge IRB in Zambia and National Health Research Authority in Lusaka, Zambia. This activity was reviewed by CDC and was conducted consistent with applicable federal law and CDC policy (See, e.g., 45 C.F.R. part 46.102(l) 21 C.F.R. part 56; 42 U.S.C. §241(d); 5 U.S.C. §552a; 44 U.S.C. §3501 et seq.). All methods were carried out in accordance with relevant guidelines and regulations. This project met requirements for waiver of informed consent documentation, as requested by the Ministry of Health, Zambia, which was granted by ERES Converge IRB in Zambia. It is embedded within the routine death registration process which legally requires the disclosure of information for vital statistics purposes. However, a verbal request is still made to the family and they have an option to opt out of participation.

## Results

A total of 67,079 VAs were conducted for deceased persons who experienced community deaths during the study period, of whom 11,475 (17.1%) were reported as persons with HIV (Table 1). Of all deaths, 47,740 (71.2%) occurred at home, 16,189 (24.1%) occurred within 24 hours of admission to a health facility, and 3,150 (4.7%) occurred in other settings (e.g., death due to motor vehicle accidents) or did not have a documented location. Among all deceased persons, a majority were male (39,251 [58.5%]). Among persons with HIV, the sex breakdown was similar, with 56.9% (6,531/11,475) male. Overall, the highest proportion of deaths occurred among persons aged ≥55 years (40.5%). The mean age of death among persons with HIV was 45 years compared to 48 years among persons without HIV (T-test p < 0.001), and <1 years for people with unknown HIV status. Lusaka (42.4%) and Copperbelt (36.9%) Provinces accounted for a majority of the deaths. Hypertension was the most commonly reported underlying medical condition among decedents (25.8%), including both persons with (22.3%) and without HIV (27.9%). For 31.0% of decedents, death was reported as occurring within 24 hours of being in regular health, with persons with HIV less likely to have died suddenly compared to persons without HIV (18.9% vs. 32.3%; p < 0.001). Collectively about half (53.8%) of decedents were recorded as having sought care at some point for the illness that led to their death. When disaggregated by HIV status, persons with HIV had a higher proportion reported to have sought care prior to death than those without HIV (62.3% vs 52.8%, p < 0.001).

**Table 1:** Characteristics and circumstances of death among deceased with verbal autopsy in Zambia, 2020-2023.

		HIV Status n (%)			
Characteristic	Overall	With HIV (N = 11475) n (%)	Without HIV (N = 51445) n (%)	Unknown (N = 4159) n (%)	P-value*
	(N = 67079) n (%)				
**Year**					
2020	15128 (22.6)	3135 (27.3)	10743 (20.9)	1250 (30.1)	**<0.001**
2021	21829 (32.5)	3561 (31.0)	16867 (32.8)	1401 (33.7)	
2022	14340 (21.4)	2274 (19.8)	11187 (21.7)	879 (21.1)	
2023	15782 (23.5%)	2505 (21.8)	12648 (24.6)	629 (15.1)	
**Sex**					
Male	39251 (58.5)	6531 (56.9)	30134 (58.6)	2586 (62.2)	**<0.001**
Female	27828 (41.5)	4944 (43.1)	21311 (41.4)	1573 (37.8)	
**Age group, years**					
<1	5971 (8.9)	140 (1.2)	3631 (7.1)	2200 (52.9)	**<0.001**
1–4	3330 (5.0)	348 (3.0)	2953 (5.7)	29 (0.7)	
5–17	3421 (5.1)	356 (3.1)	3036 (5.9)	29 (0.7)	
18–24	2989 (4.5)	462 (4.1)	2442 (4.7)	85 (2.0)	
25–34	7282 (10.9)	1790 (15.6)	5110 (9.9)	382 (9.2)	
35–44	9244 (13.8)	2869 (25.0)	5883 (11.4)	492 (11.8)	
45–54	7702 (11.5)	2321 (20.2)	5028 (9.8)	353 (8.5)	
≥55	27140 (40.5)	3189 (27.8)	23362 (45.4)	589 (14.2)	
**Province**					
Central	3141 (4.7)	588 (5.1)	2410 (4.7)	143 (3.4)	**<0.001**
Copperbelt	24772 (36.9)	3410 (29.7)	19605 (38.1)	1757 (42.2)	
Eastern	962 (1.4)	211 (1.8)	736 (1.4)	15 (0.4)	
Lusaka	28443 (42.4)	5423 (47.3)	21286 (41.4)	1734 (41.7)	
North-Western	1509 (2.3)	219 (1.9)	1244 (2.4)	46 (1.1)	
Southern	5818 (8.7)	1259 (11.0)	4253 (8.3)	306 (7.4)	
Western	2434 (3.6)	365 (3.2)	1911 (3.7)	158 (3.8)	
**Underlying conditions**					
Pregnant^†^	252 (0.9)	35 (0.7)	206 (0.9)	11 (0.7)	**<0.001**
Hypertension	17336 (25.8)	2554 (22.3)	14372 (27.9)	410 (9.9)	**<0.001**
Cardiac disease	4344 (6.5)	643 (5.6)	3609 (7.0)	92 (2.2)	**<0.001**
Diabetes	5419 (8.1)	884 (7.7)	4413 (8.6)	122 (2.9)	**<0.001**
Asthma	1903 (2.8)	325 (2.8)	1527 (3.0)	51 (1.2)	**<0.001**
Chronic obstructive pulmonary disease	1511 (2.3)	441 (3.8)	1023 (2.0)	47 (1.1)	**<0.001**
Chronic Kidney Disease	1728 (2.6)	541 (4.7)	1147 (2.2)	40 (1.0)	
					**<0.001**
Liver disease	2238 (3.3)	656 (5.7)	1517 (3.0)	65 (1.6)	**<0.001**
**Socio-behavioral Factors**					
**Marital Status** ^**#**^					
Married	22659 (41.2)	4397 (41.0)	17408 (41.2)	854 (44.6)	**<0.001**
Divorced	5826 (10.6)	1798 (16.8)	3743 (8.8)	285 (14.9)	
Partnership	147 (0.3)	42 (0.4)	94 (0.2)	11 (0.6)	
Single	11245 (20.4)	2395 (22.3)	8329 (19.7)	521 (27.1)	
Underage	308 (0.6)	45 (0.4)	259 (0.6)	4 (0.2)	
Widowed	14129 (25.7)	1906 (17.8)	12018 (28.4)	205 (10.7)	
Not Availed	674 (1.2)	144 (1.3)	495 (1.1)	35 (1.9)	
**Education Level** ^**#**^					
Not Availed	2469 (4.5)	353 (3.3)	1957 (4.6)	159 (8.3)	**<0.001**
No Formal Education	8166(14.9)	1075 (10.0)	6941 (16.4)	150 (7.8)	
Primary	21248 (38.6)	4355 (40.6)	16174 (38.2)	719 (37.5)	
Secondary	17952 (32.6)	3965 (37.0)	13294 (31.4)	693 (36.3)	
Tertiary	5153 (9.4)	979 (9.1)	3980 (9.4)	194 (10.1)	
**Economic activity** ^**#**^					
Employed	7935 (14.4)	1765 (16.5)	5731 (13.5)	439 (22.9)	
Home-Maker	2177 (4.0)	417 (3.9)	1711 (4.0)	49 (2.6)	**<0.001**
Unemployed	31361 (57.0)	6009 (56.0)	24422 (57.7)	930 (48.6)	
Pensioner	3229 (5.9)	364 (3.4)	2793 (6.6)	72 (3.8)	
Student	955 (1.7)	150 (1.4)	785 (1.9)	20 (1.0)	
Not Availed	9331 (17.0)	2022 (18.9)	6904 (16.3)	405 (21.1)	
**Recreational Drugs** ^**#**^					
Ever smoked	21844 (39.7)	4963 (46.3)	15765 (37.2)	1116 (58.3)	**<0.001**
Alcohol use	11558 (21.0)	8497 (20.1)	2504 (23.3)	557 (29.1)	**<0.001**
**Place of death**					
Home	47740 (71.2)	8550 (74.5)	36968 (71.9)	2222 (53.4)	**<0.001**
Health Facility	16189 (24.1)	2638 (23.0)	11815 (23.0)	1736 (41.7)	
Other	3150 (4.7)	287 (2.5)	2662 (5.2)	201 (4.8)	
**Died suddenly** ^**‡**^	20766 (31.0)	2164 (18.9)	16591 (32.3)	2011 (48.4)	**<0.001**
**Received care before death** ^**¶**^	36104 (53.8)	7149 (62.3)	27184 (52.8)	1771 (42.6)	**<0.001**

*Calculated using chi-square test and Fischer exact test where relevant.

^†^Analysis restricted to females.

^‡^A sudden death was defined as dying within 24 hours of being in regular/good health.

^¶^Indicates person received any form of care for the condition that led to death.

^#^Restricted to decedents aged ≥16 years (n=54988).

**Fig 1:**
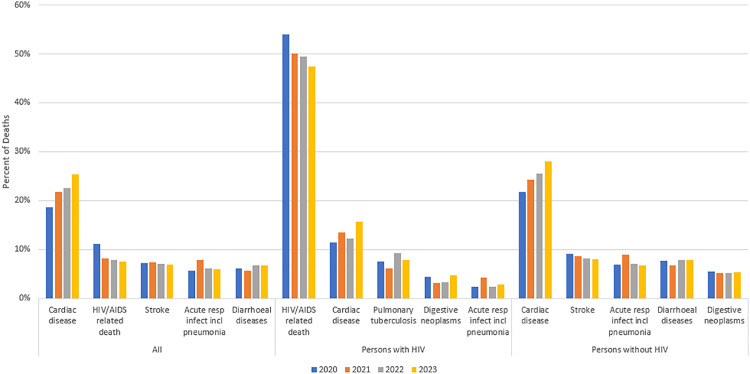
Trends in the most common verbal autopsy assigned causes of death in Zambia stratified by HIV status, 2020-2023.

Among all deceased persons, the leading CoD was cardiac disease (22.1%), and the proportion of deaths attributed to this increased from 18.7% in 2020 to 25.4% in 2023 (trend test p < 0.001; Fig 1; S1 Table in S1 File; S2A Table in S1 Table). This was followed by HIV/AIDS-related deaths (8.6%), stroke (7.1%), acute respiratory infection including pneumonia (6.5%), and diarrheal diseases (6.3%). HIV/AIDS-related deaths declined from 11.2% in 2020 to 7.5% in 2023 (p < 0.001). Deaths attributed to stroke were 7.1% to 6.9% in 2020 and 2023, respectively (p = 0.280). The proportion of deaths due to acute respiratory infection including pneumonia increased from 5.6% in 2020 to 7.8% in 2021 and then declined to 6.0% by 2023 during the COVID-19 pandemic period (p = 0.248). Diarrhea-related deaths increased from 6.1% in 2020 to 6.8% in 2023 (p < 0.001).

**Fig 2:**
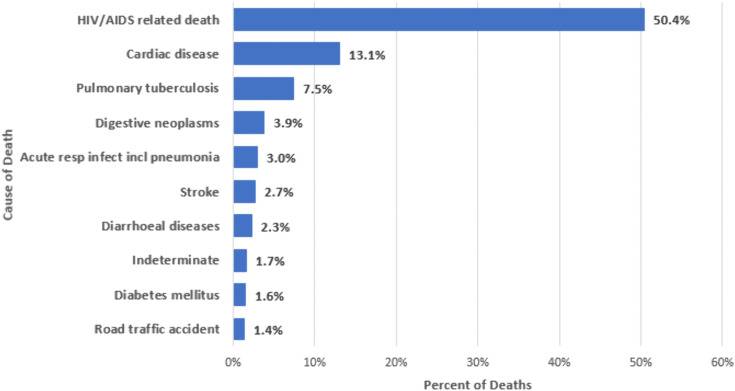
Most common verbal autopsy assigned causes of death among persons with HIV in Zambia, 2020-2023*. Abbreviations: acute resp infect incl pneumonia = acute respiratory infection including pneumonia. *Figure includes most common assigned underlying causes of death by verbal autopsy; 12.4% had another less commonly assigned cause of death.

Among persons with HIV, the most common probable CoD identified during VA were HIV/AIDS-related causes (50.4%), cardiac disease (13.1%), pulmonary tuberculosis (7.5%), digestive neoplasms (3.9%), and acute respiratory infection including pneumonia (3.0%) [(Fig 2; S1 Table in S1 File)]. From 2020 to 2023, the percentage of deaths among the leading causes of death in persons with HIV attributed to HIV/AIDS-related causes decreased from 53.9% to 47.5% (trend test p < 0.001; Fig 1; S2B Table in S1 File) whereas the percentage of deaths attributed to cardiac disease increased from 11.4% to 15.6% (p < 0.001). No significant trend over time was observed for pulmonary tuberculosis deaths (p = 0.064), digestive neoplasm-related deaths (0.565), or acute respiratory infections including pneumonia (p = 0.965).

Among decedents without HIV, the most common probable CoDs included cardiac disease (24.9%), stroke (8.5%), acute respiratory infection including pneumonia (7.6%), diarrheal disease (7.5%), and digestive neoplasms (5.2%) [Fig 1; S2C Table in S1 File]. Cardiac disease-related deaths increased from 21.7% to 28.1% in 2020 and 2023, respectively (p < 0.001). Stroke-related deaths decreased from 9.1% in 2020 to 8.1% in 2023 (p = 0.002), acute respiratory infection-related deaths increased from 6.8% in 2020 to 8.9% in 2021 and then decreased to 6.7% in 2023 (p = 0.005), whereas no significant trend was observed for diarrheal disease (p = 0.066) or digestive neoplasms (p = 0.984).

Among decedents with unknown HIV status, a majority were children (median age < 1 year) and the most common probable CoDs included prematurity (15.9%), birth asphyxia (15.5%), cardiac disease (11.8%), neonatal sepsis (5.7%), and neonatal pneumonia (5.7%) [Table 1; S1 Table in S1 File].

**Fig 3:**
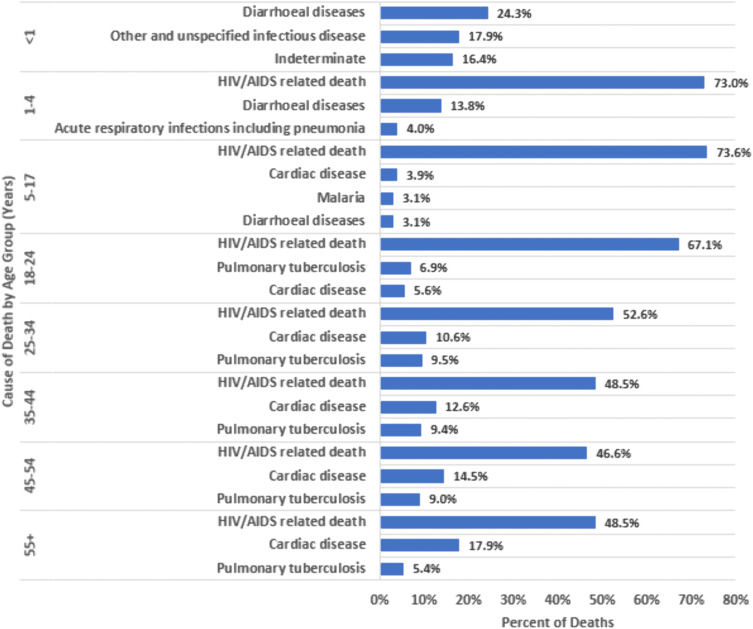
Top verbal autopsy assigned underlying causes of death in Zambia stratified by age-group among persons with HIV, 2020-2023.

By age group, among persons with HIV, HIV/AIDS-related causes accounted for the highest proportion of probable CoD for all age groups (Fig 3; S3 Table in S1 File), apart from decedents aged <1 year in whom diarrheal disease was the most common probable CoD assigned. By age strata, children aged 1–17 years had the highest proportion of deaths attributed to HIV/AIDS (i.e., 1–4 years – 73.0%, and 5–17 years – 73.6%) in comparison with the older age groups of persons with HIV.

**Fig 4:**
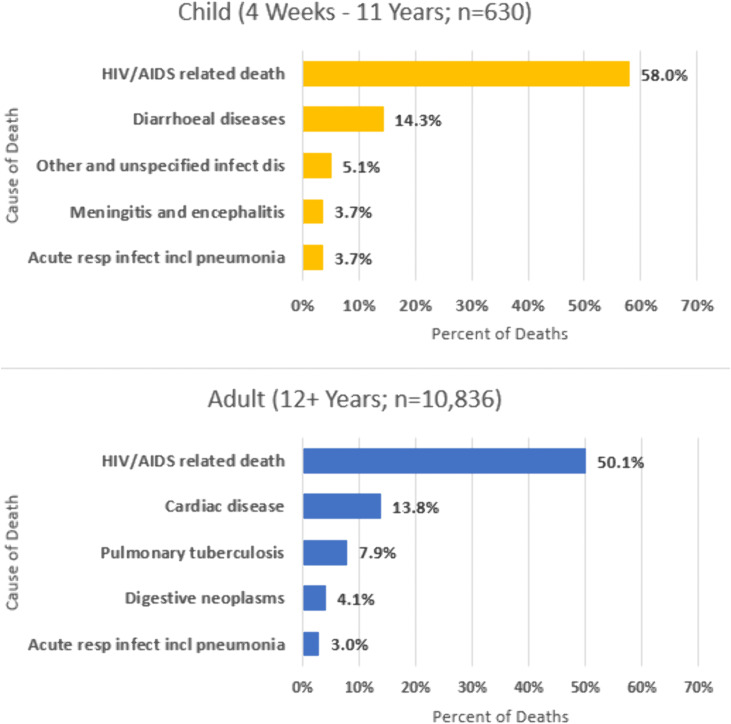
Top verbal autopsy assigned causes of death in Zambia stratified by verbal autopsy questionnaire age category among persons with HIV, 2020-2023.

When categorized by VA questionnaire type (Fig 4; S4A Table in S1 File), among persons with HIV HIV/AIDS -related CoD accounted for the most deaths as well. In the child category (4 weeks to 11 years), other leading CoDs included diarrheal diseases (14.3%), other and unspecified infections (5.1%), meningitis and encephalitis (3.7%), and acute respiratory pneumonia (3.7%). In the adult category (12 years and above), other leading CoDs for persons with HIV included cardiac disease (13.8%), pulmonary tuberculosis (7.9%), digestive neoplasms (4.1%), and acute respiratory infections including pneumonia (3.0%) [S4B Table in S1 File]. A total of 9 neonatal children with HIV were among the decedents with CoD results as follows: Indeterminate [6], prematurity [2], and neonatal pneumonia [1].

**Fig 5:**
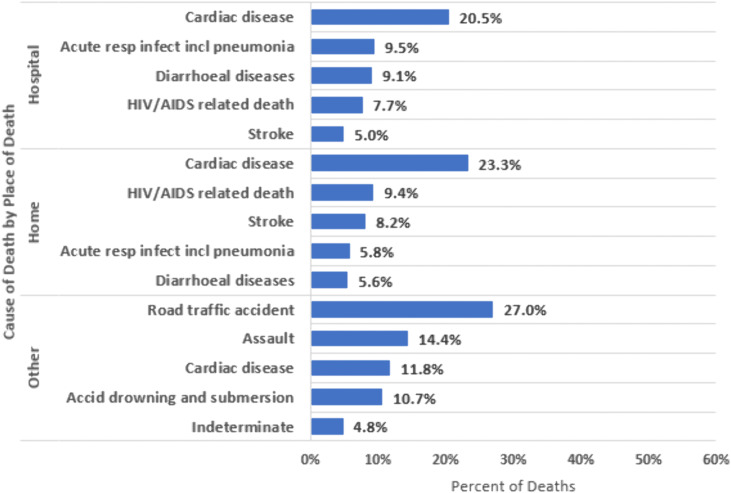
Top verbal autopsy assigned causes of death in Zambia stratified by place of death among all decedents, 2020-2023.

**Fig 6:**
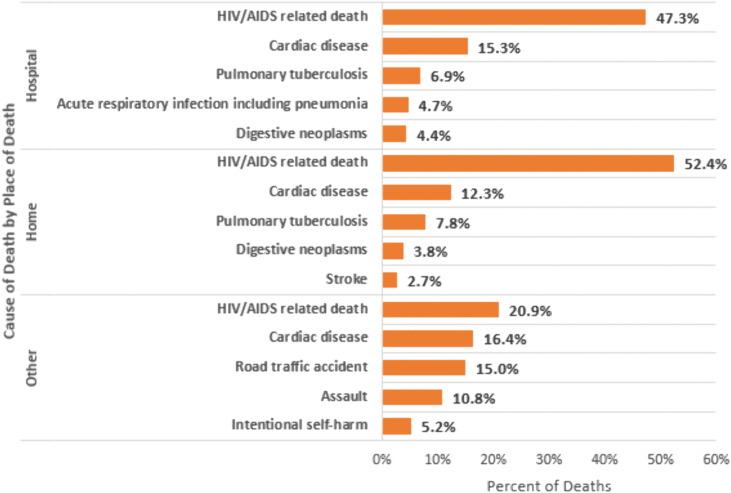
Top verbal autopsy assigned causes of death in Zambia stratified by place of death among decedents with HIV, 2020-2023.

**Fig 7:**
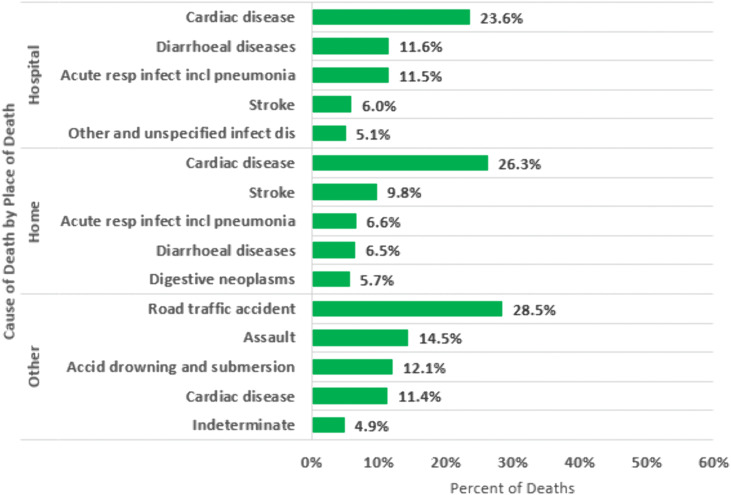
Top verbal autopsy assigned causes of death in Zambia stratified by place of death among decedents without HIV.

Stratified by place of death, probable CoD differed across settings. For all decedents (Fig 5; S5A Table in S1 File), cardiac disease was the leading cause of death at home (23.3%) and within 24 hours of arrival at a facility (20.5%), while road traffic accidents were the top cause in other or unknown locations (27.0%).Among persons with HIV (Fig 6; S5B Table in S1 File), HIV/AIDS-related causes were the leading cause of death across all locations—accounting for 52.4% of deaths at home, 47.3% of deaths within 24 hours of arriving at a facility, and 20.9% of deaths in other or unknown locations. Among persons without HIV (Fig 7; S5C Table in S1 File), cardiac disease was the leading cause of death at home (26.3%) and within 24 hours of facility arrival (23.6%). In other or unknown locations, road traffic accidents were the most common cause of death (28.5%).

## Discussion

This analysis of community mortality surveillance using verbal autopsy in Zambia identified that almost one in five decedents was a person with HIV. Amongst those both with and without HIV, CoDs included both infectious and noninfectious causes, and deaths from cardiac disease, stroke, and cancer were common. Among persons with HIV, tuberculosis was identified as the second most common cause of infectious disease-related deaths after assignment of HIV/AIDS-related causes. These findings highlight a need to strengthen ART services and retention in care for PLHIV to reduce the burden of HIV/AIDS- and TB-related deaths, as well as to identify and effectively co-manage noncommunicable conditions. Notably, about half of deceased persons with HIV had a non-specified HIV/AIDS-related cause of death assigned, suggesting that future work may be needed to better characterize specific contributors to death in this group.

In persons with HIV, a higher proportion of decedents identified through sentinel VA surveillance were male compared to the general population. This is in contrast to a prevalence of HIV in males in Zambia that is lower compared to females (adults ages 15–59 years: females 13.9% and males 8.0%) [12]. This finding may be due to late presentation to HIV treatment services for males with advanced disease, as has been identified from a previous TB study in Zambia [12,13]. These findings are also consistent with findings from other studies in sub-Saharan Africa that have found a higher prevalence of advanced HIV disease and incidence of AIDS-related conditions in men compared to women [13,14]. Collectively, these findings highlight the need to strengthen initiation and retention in HIV services for men living with HIV.

We found a high proportion of deaths overall due to noncommunicable diseases (NCDs) from VA sentinel surveillance in Zambia. This is notable, especially as the prevalence of NCDs were likely under-ascertained as VA relies on next of kin or other proxy respondents who may have incomplete knowledge of the deceased individuals’ full medical history. The high prevalence of NCD mortality underscores the need to ensure NCD service provision for all individuals in Zambia regardless of HIV status. A study by Mutale *et al.* revealed gaps in primary health care capacity to manage NCDs in Zambia, with most health facilities failing to reach the minimum threshold within 3 districts assessed. These results may be generalized to other similar districts in Zambia, where health systems remain focused on infectious diseases rather than NCDs [15]. Additionally, greater integration of services from standalone HIV care to integrated primary health care that includes NCDs management might contribute to reducing mortality among PLHIV. This integration is aligned with the Zambian guidance focused on reducing mortality for all HIV infected individuals presenting with advanced HIV disease in the country which includes NCD as an important care provision area [16].

Respiratory diseases were also frequently a cause of death. While nonspecific respiratory infections identified as a cause of death could be related to the SARS CoV-2 pandemic during the analytic period [17], tuberculosis was found to be an important contributor to the respiratory disease burden, principally in those with HIV. This finding aligns with a study by Elliot *et al.* in Zambia, which showed that at least 34% of patients with HIV for whom cause of death was known died from tuberculosis [18]. Prior studies have shown a difference in mortality between persons with HIV coinfected with tuberculosis on both antiretroviral treatment and isoniazid preventive therapy (IPT) versus those on ART alone with the latter having a higher likelihood of mortality. [19] Therefore, strengthening of strategies on IPT administration in PLHIV could be a key intervention to reduce mortality.

The high proportion of HIV/AIDS related deaths in the children highlights bigger gaps in this age group. Despite evidence suggesting the benefit of early treatment, timely identification and treatment of children remains a challenge. Better strategies for effective case finding and engagement in care are urgently needed in addition to an improved understanding of how to retain children and adolescents living with HIV on treatment [20].

Verbal autopsy was able to highlight that more than half of deaths among persons with HIV decedents were assigned as being HIV-related. However, the non-specific term, HIV-related cause of death, may not in itself be helpful for mitigation purposes because more specific contributors to death (e.g., specific opportunistic infections) were not captured within this broad category. Determination of Cause-of-Death (DeCoDe) using minimally invasive tissue sampling and other techniques could provide data to improve CoD determination. The data could subsequently be utilized to improve the CoD determination algorithms of VA and its diagnostic ability [21]. Further, verbal autopsy validation studies, that provide a framework for comparison of cause of death information from verbal autopsy methods against standard cause of death determination methods, in similar settings can help disentangle the VA findings further to contextualize information surrounding these deaths. In addition, findings can also be used to further understand the complexities around why people die from home or the community instead of seeking care for the illnesses that lead to death.

This analysis is subject to several limitations. The total number of deceased persons eligible for VA during the study period was not available, meaning the representativeness of the sampled population is unknown. Furthermore, although a large proportion of the population reside in districts where VA is conducted, approximately 46% according to the 2022 census, the findings might not be generalizable to all of Zambia. Because the VA respondent is the next-of-kin or close associate, the findings are subject to response and misclassification bias. For example, HIV status was not routinely confirmed by laboratory testing or medical records review and there is a possibility of misclassification by family members or other VA respondents who were unaware of a decedent’s HIV status or were aware but did not wish to disclose their status. This may have resulted in under-ascertainment of HIV prevalence among decedents and some HIV/AIDS-related deaths in persons with HIV being misclassified as having a CoD unrelated to HIV/AIDS. InterVA5 algorithm assigned a limited number of probable causes of death and could not subcategorize HIV/AIDS related deaths. There is a chance the algorithm could not clearly distinguish between TB and acute respiratory infections as a CoD as TB can present as acute pneumonia, and extrapulmonary TB (especially in children). In addition the causal effect of HIV/AIDS with TB on mortality could not be clearly demonstrated.

## Conclusions

Despite limitations, mortality surveillance using VA provided useful information about common causes of death among persons with HIV in Zambia. Enhanced engagement of men, including early ART initiation and retention on optimal ART regimens, may be beneficial in reducing HIV/AIDS-related deaths in this disproportionately impacted population. Given the high prevalence of mortality from NCDs, introducing interventions to improve access and strengthen integrated management of NCDs might help to reduce mortality from these conditions. Ensuring prevention and treatment of tuberculosis in HIV-infected populations could also reduce mortality. In addition, a more comprehensive analysis of validation data from complete diagnostic autopsies or physician reviews may disentangle various causes of death within the category of HIV/AIDS related death for tailored mitigation. Other strategies, such as minimally invasive tissue sampling, may also provide less resource-intensive approaches in distinguishing causes of death among persons with HIV to guide public health strategies. Finally, further investment in routine representative mortality surveillance data such as through population-representative cohorts can inform cause of death trend analyses longitudinally, help better ascertain the prevalence of underlying conditions such as malnutrition and cancer, provide a platform for monitoring public health events, and serve a role in preparing for potential epidemics. Strong vital statistics systems are critical for improving information on causes of death but, until a robust vital statistics program is established in Zambia, mortality surveillance through VA can play an important role in generating data to inform public health decision makers.

## Supporting information

S1 File Supplemental Tables. (DOCX)
