# Supplementary material for: Causes of community deaths by verbal autopsy among persons with HIV in 33 districts in Zambia, 2020–2023
Source: PLoS One. 2025 Dec 17;20(12):e0338453. doi: 10.1371/journal.pone.0338453 (PMC12711047; doi:10.1371/journal.pone.0338453)
Supplement: S1 File — (DOCX) [file pone.0338453.s001.docx]

**Supplementary Tables**:

**S1 Table. Verbal autopsy assigned causes of in Zambia stratified by HIV status, 2020-2023**

| **HIV Status** | **Person with HIV** | | **Person without HIV** | | **Unknown HIV status** | | **Overall** | |
| --- | --- | --- | --- | --- | --- | --- | --- | --- |
| **Cause of Death** | **n** | **%** | **n** | **%** | **n** | **%** | **n** | **%** |
| Cardiac disease (code 04.01 & 04.99) | 1504 | 13.1% | 12820 | 24.9% | 492 | 11.8% | 14816 | 22.1% |
| 01.03 HIV/AIDS related death | 5789 | 50.4% | 0 | 0.0% | 0 | 0.0% | 5789 | 8.6% |
| 04.02 Stroke | 315 | 2.7% | 4368 | 8.5% | 101 | 2.4% | 4784 | 7.1% |
| 01.02 Acute resp infect incl pneumonia | 346 | 3.0% | 3886 | 7.6% | 140 | 3.4% | 4372 | 6.5% |
| 01.04 Diarrhoeal diseases | 265 | 2.3% | 3839 | 7.5% | 91 | 2.2% | 4195 | 6.3% |
| 02.02 Digestive neoplasms | 445 | 3.9% | 2698 | 5.2% | 115 | 2.8% | 3258 | 4.9% |
| 01.09 Pulmonary tuberculosis | 861 | 7.5% | 1892 | 3.7% | 155 | 3.7% | 2908 | 4.3% |
| Indeterminate | 192 | 1.7% | 2260 | 4.4% | 185 | 4.4% | 2637 | 3.9% |
| 03.03 Diabetes mellitus | 183 | 1.6% | 2258 | 4.4% | 54 | 1.3% | 2495 | 3.7% |
| 12.01 Road traffic accident | 165 | 1.4% | 1818 | 3.5% | 103 | 2.5% | 2086 | 3.1% |
| 12.09 Assault | 143 | 1.2% | 1814 | 3.5% | 110 | 2.6% | 2067 | 3.1% |
| 01.99 Other and unspecified infect dis | 83 | 0.7% | 1552 | 3.0% | 44 | 1.1% | 1679 | 2.5% |
| 01.05 Malaria | 56 | 0.5% | 1223 | 2.4% | 40 | 1.0% | 1319 | 2.0% |
| 12.08 Intentional self-harm | 147 | 1.3% | 942 | 1.8% | 58 | 1.4% | 1147 | 1.7% |
| 06.02 Liver cirrhosis | 158 | 1.4% | 929 | 1.8% | 46 | 1.1% | 1133 | 1.7% |
| 01.07 Meningitis and encephalitis | 60 | 0.5% | 1027 | 2.0% | 33 | 0.8% | 1120 | 1.7% |
| 08.01 Epilepsy | 80 | 0.7% | 932 | 1.8% | 32 | 0.8% | 1044 | 1.6% |
| 02.03 Respiratory neoplasms | 132 | 1.2% | 849 | 1.7% | 43 | 1.0% | 1024 | 1.5% |
| 12.03 Accid fall | 50 | 0.4% | 656 | 1.3% | 18 | 0.4% | 724 | 1.1% |
| 02.99 Other and unspecified neoplasms | 61 | 0.5% | 617 | 1.2% | 22 | 0.5% | 700 | 1.0% |
| 10.01 Prematurity | 2 | 0.0% | 0 | 0.0% | 663 | 15.9% | 665 | 1.0% |
| 10.02 Birth asphyxia | 0 | 0.0% | 0 | 0.0% | 643 | 15.5% | 643 | 1.0% |
| 12.04 Accid drowning and submersion | 13 | 0.1% | 613 | 1.2% | 13 | 0.3% | 639 | 1.0% |
| 02.05 & 02.06 Reproductive neoplasms MF | 92 | 0.8% | 503 | 1.0% | 22 | 0.5% | 617 | 0.9% |
| 07.01 Renal failure | 50 | 0.4% | 473 | 0.9% | 17 | 0.4% | 540 | 0.8% |
| 06.01 Acute abdomen | 43 | 0.4% | 405 | 0.8% | 29 | 0.7% | 477 | 0.7% |
| 03.02 Severe malnutrition | 23 | 0.2% | 368 | 0.7% | 6 | 0.1% | 397 | 0.6% |
| 01.01 Sepsis (non-obstetric) | 35 | 0.3% | 349 | 0.7% | 3 | 0.1% | 387 | 0.6% |
| 12.99 Other and unspecified external CoD | 18 | 0.2% | 243 | 0.5% | 12 | 0.3% | 273 | 0.4% |
| 10.03 Neonatal pneumonia | 1 | 0.0% | 0 | 0.0% | 236 | 5.7% | 237 | 0.4% |
| 10.04 Neonatal sepsis | 0 | 0.0% | 0 | 0.0% | 237 | 5.7% | 237 | 0.4% |
| 12.07 Accid poisoning & noxious subs | 21 | 0.2% | 196 | 0.4% | 11 | 0.3% | 228 | 0.3% |
| 12.05 Accid expos to smoke fire & flame | 11 | 0.1% | 209 | 0.4% | 7 | 0.2% | 227 | 0.3% |
| 10.06 Congenital malformation | 4 | 0.0% | 134 | 0.3% | 87 | 2.1% | 225 | 0.3% |
| 09.04 Obstetric haemorrhage | 24 | 0.2% | 162 | 0.3% | 11 | 0.3% | 197 | 0.3% |
| 12.02 Other transport accident | 3 | 0.0% | 183 | 0.4% | 10 | 0.2% | 196 | 0.3% |
| 98 Other and unspecified NCD | 10 | 0.1% | 181 | 0.4% | 4 | 0.1% | 195 | 0.3% |
| 05.01 Chronic obstructive pulmonary dis | 6 | 0.1% | 133 | 0.3% | 4 | 0.1% | 143 | 0.2% |
| 12.10 Exposure to force of nature | 5 | 0.0% | 128 | 0.2% | 6 | 0.1% | 139 | 0.2% |
| 02.04 Breast neoplasms | 9 | 0.1% | 115 | 0.2% | 3 | 0.1% | 127 | 0.2% |
| 11.01 Fresh stillbirth | 0 | 0.0% | 0 | 0.0% | 115 | 2.8% | 115 | 0.2% |
| 02.01 Oral neoplasms | 14 | 0.1% | 75 | 0.1% | 3 | 0.1% | 92 | 0.1% |
| 11.02 Macerated stillbirth | 0 | 0.0% | 0 | 0.0% | 85 | 2.0% | 85 | 0.1% |
| 09.02 Abortion-related death | 9 | 0.1% | 71 | 0.1% | 5 | 0.1% | 85 | 0.1% |
| 05.02 Asthma | 11 | 0.1% | 71 | 0.1% | 3 | 0.1% | 85 | 0.1% |
| 01.08 & 10.05 Tetanus | 1 | 0.0% | 77 | 0.1% | 1 | 0.0% | 79 | 0.1% |
| (blank) | 5 | 0.0% | 35 | 0.1% | 28 | 0.7% | 68 | 0.1% |
| 03.01 Severe anaemia | 9 | 0.1% | 45 | 0.1% | 2 | 0.0% | 56 | 0.1% |
| 12.06 Contact with venomous plant/animal | 1 | 0.0% | 53 | 0.1% | 0 | 0.0% | 54 | 0.1% |
| 09.03 Pregnancy-induced hypertension | 5 | 0.0% | 42 | 0.1% | 2 | 0.0% | 49 | 0.1% |
| 01.10 Pertussis | 2 | 0.0% | 43 | 0.1% | 0 | 0.0% | 45 | 0.1% |
| 01.11 Haemorrhagic fever (non-dengue) | 1 | 0.0% | 38 | 0.1% | 2 | 0.0% | 41 | 0.1% |
| 01.12 Dengue fever | 3 | 0.0% | 37 | 0.1% | 0 | 0.0% | 40 | 0.1% |
| 09.06 Pregnancy-related sepsis | 4 | 0.0% | 25 | 0.0% | 3 | 0.1% | 32 | 0.0% |
| 04.03 Sickle cell with crisis | 0 | 0.0% | 17 | 0.0% | 0 | 0.0% | 17 | 0.0% |
| 09.01 Ectopic pregnancy | 0 | 0.0% | 14 | 0.0% | 0 | 0.0% | 14 | 0.0% |
| 09.99 Other and unspecified maternal CoD | 1 | 0.0% | 10 | 0.0% | 0 | 0.0% | 11 | 0.0% |
| 09.08 Ruptured uterus | 2 | 0.0% | 5 | 0.0% | 0 | 0.0% | 7 | 0.0% |
| 09.07 Anaemia of pregnancy | 2 | 0.0% | 5 | 0.0% | 0 | 0.0% | 7 | 0.0% |
| 01.06 Measles | 0 | 0.0% | 6 | 0.0% | 0 | 0.0% | 6 | 0.0% |
| 10.99 Other and unspecified neonatal CoD | 0 | 0.0% | 0 | 0.0% | 4 | 0.1% | 4 | 0.0% |
| 09.05 Obstructed labour | 0 | 0.0% | 1 | 0.0% | 0 | 0.0% | 1 | 0.0% |
| **Overall** | **11475** | **100.0%** | **51445** | **100.0%** | **4159** | **100.0%** | **67079** | **100.0%** |

**S2A Table) Trends in the verbal autopsy assigned cause of death among all deceased in Zambia, 2020-2023**

|  | **2020** | | **2021** | | **2022** | | **2023** | |
| --- | --- | --- | --- | --- | --- | --- | --- | --- |
| **Cause of Death** | **n** | **%** | **n** | **%** | **n** | **%** | **n** | **%** |
| Cardiac disease (code 04.01 & 04.99) | 2828 | 18.7% | 4740 | 21.7% | 3238 | 22.6% | 4010 | 25.4% |
| 01.03 HIV/AIDS related death | 1691 | 11.2% | 1783 | 8.2% | 1126 | 7.9% | 1189 | 7.5% |
| 04.02 Stroke | 1080 | 7.1% | 1603 | 7.3% | 1005 | 7.0% | 1096 | 6.9% |
| 01.04 Diarrhoeal diseases | 916 | 6.1% | 1242 | 5.7% | 970 | 6.8% | 1067 | 6.8% |
| 01.02 Acute respiratory infection including pneumonia | 846 | 5.6% | 1700 | 7.8% | 880 | 6.1% | 946 | 6.0% |
| 02.02 Digestive neoplasms | 763 | 5.0% | 1003 | 4.6% | 684 | 4.8% | 808 | 5.1% |
| 01.09 Pulmonary tuberculosis | 676 | 4.5% | 831 | 3.8% | 713 | 5.0% | 688 | 4.4% |
| 03.03 Diabetes mellitus | 621 | 4.1% | 1008 | 4.6% | 422 | 2.9% | 444 | 2.8% |
| Indeterminate | 599 | 4.0% | 847 | 3.9% | 642 | 4.5% | 549 | 3.5% |
| 12.09 Assault | 540 | 3.6% | 659 | 3.0% | 443 | 3.1% | 425 | 2.7% |
| 12.01 Road traffic accident | 530 | 3.5% | 764 | 3.5% | 372 | 2.6% | 420 | 2.7% |
| 01.99 Other and unspecified infectious diseases | 361 | 2.4% | 492 | 2.3% | 385 | 2.7% | 441 | 2.8% |
| 12.08 Intentional self-harm | 315 | 2.1% | 332 | 1.5% | 244 | 1.7% | 256 | 1.6% |
| 06.02 Liver cirrhosis | 261 | 1.7% | 367 | 1.7% | 238 | 1.7% | 267 | 1.7% |
| 01.07 Meningitis and encephalitis | 255 | 1.7% | 352 | 1.6% | 250 | 1.7% | 263 | 1.7% |
| 01.05 Malaria | 232 | 1.5% | 499 | 2.3% | 290 | 2.0% | 298 | 1.9% |
| 08.01 Epilepsy | 211 | 1.4% | 294 | 1.3% | 259 | 1.8% | 280 | 1.8% |
| 10.01 Prematurity | 190 | 1.3% | 254 | 1.2% | 110 | 0.8% | 111 | 0.7% |
| 10.02 Birth asphyxia | 187 | 1.2% | 239 | 1.1% | 118 | 0.8% | 99 | 0.6% |
| 02.03 Respiratory neoplasms | 179 | 1.2% | 295 | 1.4% | 241 | 1.7% | 309 | 2.0% |
| 02.99 Other and unspecified neoplasms | 169 | 1.1% | 232 | 1.1% | 150 | 1.0% | 149 | 0.9% |
| 12.03 Accidental fall | 147 | 1.0% | 205 | 0.9% | 161 | 1.1% | 211 | 1.3% |
| 07.01 Renal failure | 132 | 0.9% | 219 | 1.0% | 98 | 0.7% | 91 | 0.6% |
| 02.05 & 02.06 Reproductive neoplasms MF | 126 | 0.8% | 185 | 0.8% | 144 | 1.0% | 162 | 1.0% |
| 12.04 Accidental drowning and submersion | 116 | 0.8% | 216 | 1.0% | 145 | 1.0% | 162 | 1.0% |
| 06.01 Acute abdomen | 113 | 0.7% | 146 | 0.7% | 96 | 0.7% | 122 | 0.8% |
| 03.02 Severe malnutrition | 99 | 0.7% | 122 | 0.6% | 95 | 0.7% | 81 | 0.5% |
| 12.99 Other and unspecified external cause of death | 85 | 0.6% | 83 | 0.4% | 54 | 0.4% | 51 | 0.3% |
| 01.01 Sepsis (non-obstetric) | 76 | 0.5% | 145 | 0.7% | 78 | 0.5% | 88 | 0.6% |
| 11.01 Fresh stillbirth | 70 | 0.5% | 40 | 0.2% | 3 | 0.0% | 2 | 0.0% |
| 10.06 Congenital malformation | 63 | 0.4% | 61 | 0.3% | 48 | 0.3% | 53 | 0.3% |
| 12.05 Accidental exposure to smoke fire & flame | 61 | 0.4% | 79 | 0.4% | 46 | 0.3% | 41 | 0.3% |
| 12.07 Accidental poisoning & noxious subs | 59 | 0.4% | 68 | 0.3% | 47 | 0.3% | 54 | 0.3% |
| 10.03 Neonatal pneumonia | 57 | 0.4% | 66 | 0.3% | 51 | 0.4% | 63 | 0.4% |
| 10.04 Neonatal sepsis | 57 | 0.4% | 80 | 0.4% | 46 | 0.3% | 54 | 0.3% |
| 09.04 Obstetric haemorrhage | 48 | 0.3% | 51 | 0.2% | 55 | 0.4% | 43 | 0.3% |
| 12.10 Exposure to force of nature | 44 | 0.3% | 34 | 0.2% | 27 | 0.2% | 34 | 0.2% |
| 11.02 Macerated stillbirth | 44 | 0.3% | 40 | 0.2% |  | 0.0% | 1 | 0.0% |
| 98 Other and unspecified non-communicable diseases | 43 | 0.3% | 60 | 0.3% | 48 | 0.3% | 44 | 0.3% |
| 12.02 Other transport accident | 36 | 0.2% | 61 | 0.3% | 49 | 0.3% | 50 | 0.3% |
| 02.04 Breast neoplasms | 26 | 0.2% | 44 | 0.2% | 33 | 0.2% | 24 | 0.2% |
| 05.01 Chronic obstructive pulmonary disease | 25 | 0.2% | 48 | 0.2% | 39 | 0.3% | 31 | 0.2% |
| 02.01 Oral neoplasms | 21 | 0.1% | 26 | 0.1% | 17 | 0.1% | 28 | 0.2% |
| 09.03 Pregnancy-induced hypertension | 16 | 0.1% | 14 | 0.1% | 9 | 0.1% | 10 | 0.1% |
| 09.02 Abortion-related death | 16 | 0.1% | 30 | 0.1% | 15 | 0.1% | 24 | 0.2% |
| 01.08 & 10.05 Tetanus | 13 | 0.1% | 26 | 0.1% | 14 | 0.1% | 26 | 0.2% |
| 12.06 Contact with venomous plant/animal | 13 | 0.1% | 13 | 0.1% | 10 | 0.1% | 18 | 0.1% |
| 03.01 Severe anaemia | 12 | 0.1% | 20 | 0.1% | 14 | 0.1% | 10 | 0.1% |
| 01.11 Haemorrhagic fever (non-dengue) | 10 | 0.1% | 11 | 0.1% | 9 | 0.1% | 11 | 0.1% |
| (blank) | 10 | 0.1% | 13 | 0.1% | 35 | 0.2% | 10 | 0.1% |
| 01.10 Pertussis | 8 | 0.1% | 13 | 0.1% | 11 | 0.1% | 13 | 0.1% |
| 01.12 Dengue fever | 7 | 0.0% | 8 | 0.0% | 13 | 0.1% | 12 | 0.1% |
| 05.02 Asthma | 7 | 0.0% | 35 | 0.2% | 25 | 0.2% | 18 | 0.1% |
| 09.06 Pregnancy-related sepsis | 6 | 0.0% | 15 | 0.1% | 3 | 0.0% | 8 | 0.1% |
| 10.99 Other and unspecified neonatal cause of death | 4 | 0.0% | 0 | 0.0% | 0 | 0.0% | 0 | 0.0% |
| 09.08 Ruptured uterus | 2 | 0.0% | 2 | 0.0% | 1 | 0.0% | 2 | 0.0% |
| 09.99 Other and unspecified maternal cause of death | 2 | 0.0% | 5 | 0.0% | 3 | 0.0% | 1 | 0.0% |
| 09.07 Anaemia of pregnancy | 1 | 0.0% | 0 | 0.0% | 4 | 0.0% | 2 | 0.0% |
| 01.06 Measles | 1 | 0.0% | 0 | 0.0% | 2 | 0.0% | 3 | 0.0% |
| 04.03 Sickle cell with crisis | 1 | 0.0% | 5 | 0.0% | 6 | 0.0% | 5 | 0.0% |
| 09.01 Ectopic pregnancy | 1 | 0.0% | 4 | 0.0% | 5 | 0.0% | 4 | 0.0% |
| 09.05 Obstructed labour | 0 | 0.0% | 0 | 0.0% | 1 | 0.0% | 0 | 0.0% |
| **Overall** | **15128** | **100%** | **21829** | **100%** | **14340** | **100%** | **15782** | **100%** |

**S2B Table) Trends in verbal autopsy assigned cause of death in Zambia among persons with HIV, 2020-2023**

|  | **2020** | | **2021** | | **2022** | | **2023** | |
| --- | --- | --- | --- | --- | --- | --- | --- | --- |
| **Cause of Death** | **n** | **%** | **n** | **%** | **n** | **%** | **n** | **%** |
| 01.03 HIV/AIDS related death | 1691 | 53.9% | 1783 | 50.1% | 1126 | 49.5% | 1189 | 47.5% |
| Cardiac disease (code 04.01 & 04.99) | 357 | 11.4% | 479 | 13.5% | 277 | 12.2% | 391 | 15.6% |
| 01.09 Pulmonary tuberculosis | 234 | 7.5% | 220 | 6.2% | 211 | 9.3% | 196 | 7.8% |
| 02.02 Digestive neoplasms | 138 | 4.4% | 113 | 3.2% | 76 | 3.3% | 118 | 4.7% |
| 04.02 Stroke | 75 | 2.4% | 116 | 3.3% | 56 | 2.5% | 68 | 2.7% |
| 01.02 Acute resp infect incl pneumonia | 72 | 2.3% | 148 | 4.2% | 54 | 2.4% | 72 | 2.9% |
| 01.04 Diarrhoeal diseases | 66 | 2.1% | 82 | 2.3% | 63 | 2.8% | 54 | 2.2% |
| 03.03 Diabetes mellitus | 53 | 1.7% | 71 | 2.0% | 31 | 1.4% | 28 | 1.1% |
| Indeterminate | 53 | 1.7% | 55 | 1.5% | 43 | 1.9% | 41 | 1.6% |
| 12.08 Intentional self-harm | 50 | 1.6% | 43 | 1.2% | 31 | 1.4% | 23 | 0.9% |
| 06.02 Liver cirrhosis | 42 | 1.3% | 57 | 1.6% | 25 | 1.1% | 34 | 1.4% |
| 12.09 Assault | 35 | 1.1% | 45 | 1.3% | 22 | 1.0% | 41 | 1.6% |
| 12.01 Road traffic accident | 33 | 1.1% | 54 | 1.5% | 32 | 1.4% | 46 | 1.8% |
| 01.99 Other and unspecified infect dis | 31 | 1.0% | 16 | 0.4% | 21 | 0.9% | 15 | 0.6% |
| 08.01 Epilepsy | 23 | 0.7% | 21 | 0.6% | 21 | 0.9% | 15 | 0.6% |
| 02.03 Respiratory neoplasms | 23 | 0.7% | 32 | 0.9% | 33 | 1.5% | 44 | 1.8% |
| 02.05 & 02.06 Reproductive neoplasms MF | 22 | 0.7% | 22 | 0.6% | 25 | 1.1% | 23 | 0.9% |
| 01.07 Meningitis and encephalitis | 16 | 0.5% | 21 | 0.6% | 13 | 0.6% | 10 | 0.4% |
| 02.99 Other and unspecified neoplasms | 16 | 0.5% | 20 | 0.6% | 14 | 0.6% | 11 | 0.4% |
| 06.01 Acute abdomen | 15 | 0.5% | 12 | 0.3% | 7 | 0.3% | 9 | 0.4% |
| 01.05 Malaria | 12 | 0.4% | 29 | 0.8% | 8 | 0.4% | 7 | 0.3% |
| 12.03 Accid fall | 10 | 0.3% | 14 | 0.4% | 15 | 0.7% | 11 | 0.4% |
| 03.02 Severe malnutrition | 10 | 0.3% | 6 | 0.2% | 5 | 0.2% | 2 | 0.1% |
| 07.01 Renal failure | 9 | 0.3% | 25 | 0.7% | 8 | 0.4% | 8 | 0.3% |
| 12.99 Other and unspecified external CoD | 7 | 0.2% | 5 | 0.1% | 1 | 0.0% | 5 | 0.2% |
| 09.04 Obstetric haemorrhage | 6 | 0.2% | 4 | 0.1% | 10 | 0.4% | 4 | 0.2% |
| 12.04 Accid drowning and submersion | 4 | 0.1% | 4 | 0.1% | 3 | 0.1% | 2 | 0.1% |
| 01.01 Sepsis (non-obstetric) | 4 | 0.1% | 20 | 0.6% | 3 | 0.1% | 8 | 0.3% |
| 12.05 Accid expos to smoke fire & flame | 3 | 0.1% | 4 | 0.1% | 3 | 0.1% | 1 | 0.0% |
| 12.07 Accid poisoning & noxious subs | 3 | 0.1% | 6 | 0.2% | 8 | 0.4% | 4 | 0.2% |
| 09.02 Abortion-related death | 3 | 0.1% | 4 | 0.1% |  | 0.0% | 2 | 0.1% |
| 98 Other and unspecified NCD | 3 | 0.1% | 4 | 0.1% | 1 | 0.0% | 2 | 0.1% |
| 09.03 Pregnancy-induced hypertension | 2 | 0.1% | 1 | 0.0% | 1 | 0.0% | 1 | 0.0% |
| 02.01 Oral neoplasms | 2 | 0.1% | 2 | 0.1% | 4 | 0.2% | 6 | 0.2% |
| 09.06 Pregnancy-related sepsis | 2 | 0.1% | 2 | 0.1% | 0 | 0.0% | 0 | 0.0% |
| 03.01 Severe anaemia | 2 | 0.1% | 5 | 0.1% | 2 | 0.1% | 0 | 0.0% |
| 05.01 Chronic obstructive pulmonary dis | 1 | 0.0% | 0 | 0.0% | 5 | 0.2% | 0 | 0.0% |
| 10.03 Neonatal pneumonia | 1 | 0.0% | 0 | 0.0% | 0 | 0.0% | 0 | 0.0% |
| 05.02 Asthma | 1 | 0.0% | 5 | 0.1% | 2 | 0.1% | 3 | 0.1% |
| 10.06 Congenital malformation | 1 | 0.0% | 0 | 0.0% | 2 | 0.1% | 1 | 0.0% |
| 01.11 Haemorrhagic fever (non-dengue) | 1 | 0.0% | 0 | 0.0% | 0 | 0.0% | 0 | 0.0% |
| 02.04 Breast neoplasms | 1 | 0.0% | 4 | 0.1% | 0 | 0.0% | 4 | 0.2% |
| 01.08 & 10.05 Tetanus | 1 | 0.0% | 0 | 0.0% | 0 | 0.0% | 0 | 0.0% |
| Missing | 1 | 0.0% | 3 | 0.1% | 1 | 0.0% | 0 | 0.0% |
| 12.02 Other transport accident | 0 | 0.0% | 0 | 0.0% | 1 | 0.0% | 2 | 0.1% |
| 10.01 Prematurity | 0 | 0.0% | 1 | 0.0% | 1 | 0.0% | 0 | 0.0% |
| 09.99 Other and unspecified maternal CoD | 0 | 0.0% | 0 | 0.0% | 1 | 0.0% | 0 | 0.0% |
| 01.10 Pertussis | 0 | 0.0% | 1 | 0.0% | 1 | 0.0% | 0 | 0.0% |
| 01.12 Dengue fever | 0 | 0.0% | 1 | 0.0% | 1 | 0.0% | 1 | 0.0% |
| 09.07 Anaemia of pregnancy | 0 | 0.0% | 0 | 0.0% | 2 | 0.1% | 0 | 0.0% |
| 12.10 Exposure to force of nature | 0 | 0.0% | 0 | 0.0% | 3 | 0.1% | 2 | 0.1% |
| 12.06 Contact with venomous plant/animal | 0 | 0.0% | 0 | 0.0% | 1 | 0.0% | 0 | 0.0% |
| 09.08 Ruptured uterus | 0 | 0.0% | 1 | 0.0% | 0 | 0.0% | 1 | 0.0% |
| **Overall** | **3135** | **100%** | **3561** | **100%** | **2274** | **100%** | **2505** | **100%** |

**S2C Table) Trends in verbal autopsy assigned cause of death in Zambia among persons without HIV, 2020-2023**

|  | **2020** | | **2021** | | **2022** | | **2023** | |
| --- | --- | --- | --- | --- | --- | --- | --- | --- |
| **Cause of Death** | **n** | **%** | **n** | **%** | **n** | **%** | **n** | **%** |
| Cardiac disease (code 04.01 & 04.99) | 2334 | 21.7% | 4091 | 24.3% | 2847 | 25.4% | 3548 | 28.1% |
| 04.02 Stroke | 976 | 9.1% | 1456 | 8.6% | 917 | 8.2% | 1019 | 8.1% |
| 01.04 Diarrhoeal diseases | 828 | 7.7% | 1139 | 6.8% | 877 | 7.8% | 995 | 7.9% |
| 01.02 Acute resp infect incl pneumonia | 733 | 6.8% | 1509 | 8.9% | 793 | 7.1% | 851 | 6.7% |
| 02.02 Digestive neoplasms | 581 | 5.4% | 859 | 5.1% | 584 | 5.2% | 674 | 5.3% |
| 03.03 Diabetes mellitus | 547 | 5.1% | 918 | 5.4% | 382 | 3.4% | 411 | 3.2% |
| Indeterminate | 496 | 4.6% | 731 | 4.3% | 553 | 4.9% | 480 | 3.8% |
| 12.09 Assault | 476 | 4.4% | 571 | 3.4% | 392 | 3.5% | 375 | 3.0% |
| 12.01 Road traffic accident | 460 | 4.3% | 673 | 4.0% | 322 | 2.9% | 363 | 2.9% |
| 01.09 Pulmonary tuberculosis | 402 | 3.7% | 564 | 3.3% | 460 | 4.1% | 466 | 3.7% |
| 01.99 Other and unspecified infect dis | 312 | 2.9% | 466 | 2.8% | 352 | 3.1% | 422 | 3.3% |
| 12.08 Intentional self-harm | 245 | 2.3% | 270 | 1.6% | 203 | 1.8% | 224 | 1.8% |
| 01.07 Meningitis and encephalitis | 227 | 2.1% | 323 | 1.9% | 229 | 2.0% | 248 | 2.0% |
| 01.05 Malaria | 214 | 2.0% | 456 | 2.7% | 272 | 2.4% | 281 | 2.2% |
| 06.02 Liver cirrhosis | 206 | 1.9% | 296 | 1.8% | 199 | 1.8% | 228 | 1.8% |
| 08.01 Epilepsy | 179 | 1.7% | 260 | 1.5% | 229 | 2.0% | 264 | 2.1% |
| 02.99 Other and unspecified neoplasms | 147 | 1.4% | 206 | 1.2% | 132 | 1.2% | 132 | 1.0% |
| 02.03 Respiratory neoplasms | 143 | 1.3% | 255 | 1.5% | 191 | 1.7% | 260 | 2.1% |
| 12.03 Accid fall | 130 | 1.2% | 189 | 1.1% | 137 | 1.2% | 200 | 1.6% |
| 07.01 Renal failure | 116 | 1.1% | 187 | 1.1% | 87 | 0.8% | 83 | 0.7% |
| 12.04 Accid drowning and submersion | 110 | 1.0% | 201 | 1.2% | 142 | 1.3% | 160 | 1.3% |
| 02.05 & 02.06 Reproductive neoplasms MF | 98 | 0.9% | 155 | 0.9% | 114 | 1.0% | 136 | 1.1% |
| 06.01 Acute abdomen | 91 | 0.8% | 130 | 0.8% | 78 | 0.7% | 106 | 0.8% |
| 03.02 Severe malnutrition | 88 | 0.8% | 116 | 0.7% | 87 | 0.8% | 77 | 0.6% |
| 12.99 Other and unspecified external CoD | 74 | 0.7% | 75 | 0.4% | 50 | 0.4% | 44 | 0.3% |
| 01.01 Sepsis (non-obstetric) | 71 | 0.7% | 124 | 0.7% | 74 | 0.7% | 80 | 0.6% |
| 12.07 Accid poisoning & noxious subs | 54 | 0.5% | 58 | 0.3% | 35 | 0.3% | 49 | 0.4% |
| 12.05 Accid expos to smoke fire & flame | 54 | 0.5% | 73 | 0.4% | 43 | 0.4% | 39 | 0.3% |
| 98 Other and unspecified NCD | 40 | 0.4% | 54 | 0.3% | 45 | 0.4% | 42 | 0.3% |
| 09.04 Obstetric haemorrhage | 40 | 0.4% | 43 | 0.3% | 41 | 0.4% | 38 | 0.3% |
| 12.10 Exposure to force of nature | 39 | 0.4% | 34 | 0.2% | 23 | 0.2% | 32 | 0.3% |
| 12.02 Other transport accident | 33 | 0.3% | 55 | 0.3% | 47 | 0.4% | 48 | 0.4% |
| 10.06 Congenital malformation | 31 | 0.3% | 38 | 0.2% | 29 | 0.3% | 36 | 0.3% |
| 02.04 Breast neoplasms | 25 | 0.2% | 39 | 0.2% | 31 | 0.3% | 20 | 0.2% |
| 05.01 Chronic obstructive pulmonary dis | 23 | 0.2% | 48 | 0.3% | 31 | 0.3% | 31 | 0.2% |
| 02.01 Oral neoplasms | 18 | 0.2% | 24 | 0.1% | 12 | 0.1% | 21 | 0.2% |
| 09.03 Pregnancy-induced hypertension | 13 | 0.1% | 12 | 0.1% | 8 | 0.1% | 9 | 0.1% |
| 12.06 Contact with venomous plant/animal | 13 | 0.1% | 13 | 0.1% | 9 | 0.1% | 18 | 0.1% |
| 01.08 & 10.05 Tetanus | 12 | 0.1% | 25 | 0.1% | 14 | 0.1% | 26 | 0.2% |
| 09.02 Abortion-related death | 11 | 0.1% | 23 | 0.1% | 15 | 0.1% | 22 | 0.2% |
| 03.01 Severe anaemia | 9 | 0.1% | 15 | 0.1% | 12 | 0.1% | 9 | 0.1% |
| 01.10 Pertussis | 8 | 0.1% | 12 | 0.1% | 10 | 0.1% | 13 | 0.1% |
| 01.12 Dengue fever | 7 | 0.1% | 7 | 0.0% | 12 | 0.1% | 11 | 0.1% |
| 01.11 Haemorrhagic fever (non-dengue) | 7 | 0.1% | 11 | 0.1% | 9 | 0.1% | 11 | 0.1% |
| 05.02 Asthma | 6 | 0.1% | 29 | 0.2% | 22 | 0.2% | 14 | 0.1% |
| (blank) | 5 | 0.0% | 8 | 0.0% | 14 | 0.1% | 8 | 0.1% |
| 09.06 Pregnancy-related sepsis | 3 | 0.0% | 11 | 0.1% | 3 | 0.0% | 8 | 0.1% |
| 09.08 Ruptured uterus | 2 | 0.0% | 1 | 0.0% | 1 | 0.0% | 1 | 0.0% |
| 09.99 Other and unspecified maternal CoD | 2 | 0.0% | 5 | 0.0% | 2 | 0.0% | 1 | 0.0% |
| 09.01 Ectopic pregnancy | 1 | 0.0% | 4 | 0.0% | 5 | 0.0% | 4 | 0.0% |
| 01.06 Measles | 1 | 0.0% | 0 | 0.0% | 2 | 0.0% | 3 | 0.0% |
| 09.07 Anaemia of pregnancy | 1 | 0.0% | 0 | 0.0% | 2 | 0.0% | 2 | 0.0% |
| 04.03 Sickle cell with crisis | 1 | 0.0% | 5 | 0.0% | 6 | 0.1% | 5 | 0.0% |
| 09.05 Obstructed labour | 0 | 0.0% | 0 | 0.0% | 1 | 0.0% | 0 | 0.0% |
| **Overall** | **10743** | **100%** | **16867** | **100%** | **11187** | **100%** | **12648** | **100%** |

**S3 Table) Verbal autopsy assigned causes of death in Zambia stratified by age-group among persons with HIV, 2020-2023**

| **Cause of Death** | **Age Group (Years)** | | | | | | | | |
| --- | --- | --- | --- | --- | --- | --- | --- | --- | --- |
|  | **<1** | **1 to 4** | **5 to 17** | **18 to 24** | **25 to 34** | **35 to 44** | **45 to 54** | **55+** | **All Ages** |
| 01.03 HIV/AIDS related death | 3 (2.1%) | 254 (73.0%) | 262 (73.6%) | 310 (67.1%) | 941 (52.6%) | 1391 (48.5%) | 1082 (46.6%) | 1546 (48.5%) | 5789 (50.4%) |
| Cardiac disease (code 04.01 & 04.99) | 5 (3.6%) | 0 (0.0%) | 14 (3.9%) | 26 (5.6%) | 189 (10.6%) | 362 (12.6%) | 336 (14.5%) | 572 (17.9%) | 1504 (13.1%) |
| 01.09 Pulmonary tuberculosis | 0 (0.0%) | 1 (0.3%) | 7 (2.0%) | 32 (6.9%) | 170 (9.5%) | 269 (9.4%) | 210 (9.0%) | 172 (5.4%) | 861 (7.5%) |
| 02.02 Digestive neoplasms | 0 (0.0%) | 0 (0.0%) | 2 (0.6%) | 12 (2.6%) | 67 (3.7%) | 108 (3.8%) | 121 (5.2%) | 135 (4.2%) | 445 (3.9%) |
| 01.02 Acute resp infect incl pneumonia | 3 (2.1%) | 14 (4.0%) | 9 (2.5%) | 6 (1.3%) | 52 (2.9%) | 85 (3.0%) | 77 (3.3%) | 100 (3.1%) | 346 (3.0%) |
| 04.02 Stroke | 0 (0.0%) | 0 (0.0%) | 1 (0.3%) | 2 (0.4%) | 17 (0.9%) | 56 (2.0%) | 70 (3.0%) | 169 (5.3%) | 315 (2.7%) |
| 01.04 Diarrhoeal diseases | 34 (24.3%) | 48 (13.8%) | 11 (3.1%) | 6 (1.3%) | 36 (2.0%) | 53 (1.8%) | 37 (1.6%) | 40 (1.3%) | 265 (2.3%) |
| Indeterminate | 23 (16.4%) | 1 (0.3%) | 0 (0.0%) | 12 (2.6%) | 34 (1.9%) | 55 (1.9%) | 33 (1.4%) | 34 (1.1%) | 192 (1.7%) |
| 03.03 Diabetes mellitus | 0 (0.0%) | 1 (0.3%) | 0 (0.0%) | 2 (0.4%) | 19 (1.1%) | 22 (0.8%) | 46 (2.0%) | 93 (2.9%) | 183 (1.6%) |
| 12.01 Road traffic accident | 0 (0.0%) | 3 (0.9%) | 3 (0.8%) | 8 (1.7%) | 29 (1.6%) | 50 (1.7%) | 41 (1.8%) | 31 (1.0%) | 165 (1.4%) |
| 06.02 Liver cirrhosis | 0 (0.0%) | 1 (0.3%) | 4 (1.1%) | 0 (0.0%) | 21 (1.2%) | 40 (1.4%) | 27 (1.2%) | 65 (2.0%) | 158 (1.4%) |
| 12.08 Intentional self-harm | 0 (0.0%) | 0 (0.0%) | 0 (0.0%) | 13 (2.8%) | 40 (2.2%) | 54 (1.9%) | 26 (1.1%) | 14 (0.4%) | 147 (1.3%) |
| 12.09 Assault | 0 (0.0%) | 0 (0.0%) | 2 (0.6%) | 7 (1.5%) | 28 (1.6%) | 66 (2.3%) | 38 (1.6%) | 2 (0.1%) | 143 (1.2%) |
| 02.03 Respiratory neoplasms | 0 (0.0%) | 0 (0.0%) | 0 (0.0%) | 2 (0.4%) | 19 (1.1%) | 51 (1.8%) | 30 (1.3%) | 30 (0.9%) | 132 (1.2%) |
| 02.05 & 02.06 Reproductive neoplasms MF | 0 (0.0%) | 0 (0.0%) | 0 (0.0%) | 0 (0.0%) | 14 (0.8%) | 31 (1.1%) | 34 (1.5%) | 13 (0.4%) | 92 (0.8%) |
| 01.99 Other and unspecified infect dis | 25 (17.9%) | 3 (0.9%) | 9 (2.5%) | 1 (0.2%) | 9 (0.5%) | 18 (0.6%) | 9 (0.4%) | 9 (0.3%) | 83 (0.7%) |
| 08.01 Epilepsy | 4 (2.9%) | 2 (0.6%) | 5 (1.4%) | 7 (1.5%) | 10 (0.6%) | 27 (0.9%) | 14 (0.6%) | 11 (0.3%) | 80 (0.7%) |
| 02.99 Other and unspecified neoplasms | 0 (0.0%) | 0 (0.0%) | 0 (0.0%) | 0 (0.0%) | 7 (0.4%) | 14 (0.5%) | 12 (0.5%) | 28 (0.9%) | 61 (0.5%) |
| 01.07 Meningitis and encephalitis | 20 (14.3%) | 2 (0.6%) | 3 (0.8%) | 0 (0.0%) | 6 (0.3%) | 14 (0.5%) | 13 (0.6%) | 2 (0.1%) | 60 (0.5%) |
| 01.05 Malaria | 0 (0.0%) | 2 (0.6%) | 11 (3.1%) | 1 (0.2%) | 6 (0.3%) | 12 (0.4%) | 7 (0.3%) | 17 (0.5%) | 56 (0.5%) |
| 12.03 Accid fall | 0 (0.0%) | 1 (0.3%) | 2 (0.6%) | 1 (0.2%) | 3 (0.2%) | 11 (0.4%) | 6 (0.3%) | 26 (0.8%) | 50 (0.4%) |
| 07.01 Renal failure | 1 (0.7%) | 0 (0.0%) | 1 (0.3%) | 0 (0.0%) | 4 (0.2%) | 9 (0.3%) | 9 (0.4%) | 26 (0.8%) | 50 (0.4%) |
| 06.01 Acute abdomen | 0 (0.0%) | 0 (0.0%) | 3 (0.8%) | 1 (0.2%) | 12 (0.7%) | 6 (0.2%) | 10 (0.4%) | 11 (0.3%) | 43 (0.4%) |
| 01.01 Sepsis (non-obstetric) | 0 (0.0%) | 2 (0.6%) | 0 (0.0%) | 0 (0.0%) | 9 (0.5%) | 12 (0.4%) | 5 (0.2%) | 7 (0.2%) | 35 (0.3%) |
| 09.04 Obstetric haemorrhage | 0 (0.0%) | 0 (0.0%) | 0 (0.0%) | 3 (0.6%) | 10 (0.6%) | 9 (0.3%) | 2 (0.1%) | 0 (0.0%) | 24 (0.2%) |
| 03.02 Severe malnutrition | 11 (7.9%) | 6 (1.7%) | 4 (1.1%) | 0 (0.0%) | 0 (0.0%) | 0 (0.0%) | 0 (0.0%) | 2 (0.1%) | 23 (0.2%) |
| 12.07 Accid poisoning & noxious subs | 0 (0.0%) | 0 (0.0%) | 0 (0.0%) | 1 (0.2%) | 8 (0.4%) | 6 (0.2%) | 5 (0.2%) | 1 (0.0%) | 21 (0.2%) |
| 12.99 Other and unspecified external CoD | 0 (0.0%) | 0 (0.0%) | 0 (0.0%) | 0 (0.0%) | 5 (0.3%) | 4 (0.1%) | 8 (0.3%) | 1 (0.0%) | 18 (0.2%) |
| 02.01 Oral neoplasms | 0 (0.0%) | 0 (0.0%) | 0 (0.0%) | 2 (0.4%) | 1 (0.1%) | 6 (0.2%) | 1 (0.0%) | 4 (0.1%) | 14 (0.1%) |
| 12.04 Accid drowning and submersion | 1 (0.7%) | 3 (0.9%) | 0 (0.0%) | 1 (0.2%) | 5 (0.3%) | 2 (0.1%) | 0 (0.0%) | 1 (0.0%) | 13 (0.1%) |
| 05.02 Asthma | 0 (0.0%) | 0 (0.0%) | 0 (0.0%) | 1 (0.2%) | 2 (0.1%) | 2 (0.1%) | 0 (0.0%) | 6 (0.2%) | 11 (0.1%) |
| 12.05 Accid expos to smoke fire & flame | 1 (0.7%) | 1 (0.3%) | 0 (0.0%) | 0 (0.0%) | 0 (0.0%) | 5 (0.2%) | 0 (0.0%) | 4 (0.1%) | 11 (0.1%) |
| 98 Other and unspecified NCD | 0 (0.0%) | 0 (0.0%) | 1 (0.3%) | 0 (0.0%) | 0 (0.0%) | 3 (0.1%) | 3 (0.1%) | 3 (0.1%) | 10 (0.1%) |
| 09.02 Abortion-related death | 0 (0.0%) | 0 (0.0%) | 0 (0.0%) | 1 (0.2%) | 4 (0.2%) | 4 (0.1%) | 0 (0.0%) | 0 (0.0%) | 9 (0.1%) |
| 03.01 Severe anaemia | 0 (0.0%) | 0 (0.0%) | 0 (0.0%) | 0 (0.0%) | 2 (0.1%) | 0 (0.0%) | 0 (0.0%) | 7 (0.2%) | 9 (0.1%) |
| 02.04 Breast neoplasms | 0 (0.0%) | 0 (0.0%) | 0 (0.0%) | 0 (0.0%) | 1 (0.1%) | 3 (0.1%) | 3 (0.1%) | 2 (0.1%) | 9 (0.1%) |
| 05.01 Chronic obstructive pulmonary dis | 0 (0.0%) | 0 (0.0%) | 0 (0.0%) | 0 (0.0%) | 0 (0.0%) | 1 (0.0%) | 1 (0.0%) | 4 (0.1%) | 6 (0.1%) |
| 12.10 Exposure to force of nature | 0 (0.0%) | 0 (0.0%) | 0 (0.0%) | 0 (0.0%) | 1 (0.1%) | 3 (0.1%) | 0 (0.0%) | 1 (0.0%) | 5 (0.0%) |
| 09.03 Pregnancy-induced hypertension | 0 (0.0%) | 0 (0.0%) | 0 (0.0%) | 0 (0.0%) | 3 (0.2%) | 2 (0.1%) | 0 (0.0%) | 0 (0.0%) | 5 (0.0%) |
| (blank) | 1 (0.7%) | 0 (0.0%) | 0 (0.0%) | 0 (0.0%) | 1 (0.1%) | 1 (0.0%) | 2 (0.1%) | 0 (0.0%) | 5 (0.0%) |
| 09.06 Pregnancy-related sepsis | 0 (0.0%) | 0 (0.0%) | 1 (0.3%) | 1 (0.2%) | 1 (0.1%) | 1 (0.0%) | 0 (0.0%) | 0 (0.0%) | 4 (0.0%) |
| 10.06 Congenital malformation | 4 (2.9%) | 0 (0.0%) | 0 (0.0%) | 0 (0.0%) | 0 (0.0%) | 0 (0.0%) | 0 (0.0%) | 0 (0.0%) | 4 (0.0%) |
| 12.02 Other transport accident | 0 (0.0%) | 0 (0.0%) | 0 (0.0%) | 1 (0.2%) | 0 (0.0%) | 1 (0.0%) | 1 (0.0%) | 0 (0.0%) | 3 (0.0%) |
| 01.12 Dengue fever | 0 (0.0%) | 1 (0.3%) | 1 (0.3%) | 0 (0.0%) | 0 (0.0%) | 0 (0.0%) | 1 (0.0%) | 0 (0.0%) | 3 (0.0%) |
| 09.08 Ruptured uterus | 0 (0.0%) | 0 (0.0%) | 0 (0.0%) | 0 (0.0%) | 1 (0.1%) | 0 (0.0%) | 1 (0.0%) | 0 (0.0%) | 2 (0.0%) |
| 10.01 Prematurity | 2 (1.4%) | 0 (0.0%) | 0 (0.0%) | 0 (0.0%) | 0 (0.0%) | 0 (0.0%) | 0 (0.0%) | 0 (0.0%) | 2 (0.0%) |
| 01.10 Pertussis | 0 (0.0%) | 2 (0.6%) | 0 (0.0%) | 0 (0.0%) | 0 (0.0%) | 0 (0.0%) | 0 (0.0%) | 0 (0.0%) | 2 (0.0%) |
| 09.07 Anaemia of pregnancy | 0 (0.0%) | 0 (0.0%) | 0 (0.0%) | 0 (0.0%) | 2 (0.1%) | 0 (0.0%) | 0 (0.0%) | 0 (0.0%) | 2 (0.0%) |
| 09.99 Other and unspecified maternal CoD | 0 (0.0%) | 0 (0.0%) | 0 (0.0%) | 1 (0.2%) | 0 (0.0%) | 0 (0.0%) | 0 (0.0%) | 0 (0.0%) | 1 (0.0%) |
| 01.08 & 10.05 Tetanus | 1 (0.7%) | 0 (0.0%) | 0 (0.0%) | 0 (0.0%) | 0 (0.0%) | 0 (0.0%) | 0 (0.0%) | 0 (0.0%) | 1 (0.0%) |
| 12.06 Contact with venomous plant/animal | 0 (0.0%) | 0 (0.0%) | 0 (0.0%) | 0 (0.0%) | 1 (0.1%) | 0 (0.0%) | 0 (0.0%) | 0 (0.0%) | 1 (0.0%) |
| 10.03 Neonatal pneumonia | 1 (0.7%) | 0 (0.0%) | 0 (0.0%) | 0 (0.0%) | 0 (0.0%) | 0 (0.0%) | 0 (0.0%) | 0 (0.0%) | 1 (0.0%) |
| 01.11 Haemorrhagic fever (non-dengue) | 0 (0.0%) | 0 (0.0%) | 0 (0.0%) | 1 (0.2%) | 0 (0.0%) | 0 (0.0%) | 0 (0.0%) | 0 (0.0%) | 1 (0.0%) |
| **Overall** | **140 (1.2%)** | **348 (3.1%)** | **356 (3.1%)** | **462 (4.0%)** | **1790 (15.6%)** | **2869 (25.0%)** | **2321 (20.2%)** | **3189 (27.8%)** | **11475 (100%)** |

**S4A Table) Verbal autopsy assigned causes of death in Zambia among persons living with HIV aged 4 weeks to 11 years, 2020-2023**

| **Cause of Death** | **n** | **%** |
| --- | --- | --- |
| 01.03 HIV/AIDS related death | 365 | 57.9% |
| 01.04 Diarrhoeal diseases | 90 | 14.3% |
| 01.99 Other and unspecified infect dis | 32 | 5.1% |
| 01.07 Meningitis and encephalitis | 23 | 3.7% |
| 01.02 Acute resp infect incl pneumonia | 23 | 3.7% |
| 03.02 Severe malnutrition | 21 | 3.3% |
| Indeterminate | 18 | 2.9% |
| 01.05 Malaria | 12 | 1.9% |
| 08.01 Epilepsy | 7 | 1.1% |
| Cardiac disease (code 04.01 & 04.99) | 7 | 1.1% |
| 12.01 Road traffic accident | 5 | 0.8% |
| 12.04 Accid drowning and submersion | 4 | 0.6% |
| 10.06 Congenital malformation | 4 | 0.6% |
| 01.09 Pulmonary tuberculosis | 3 | 0.5% |
| 01.10 Pertussis | 2 | 0.3% |
| 12.05 Accid expos to smoke fire & flame | 2 | 0.3% |
| 01.12 Dengue fever | 2 | 0.3% |
| 01.01 Sepsis (non-obstetric) | 2 | 0.3% |
| 07.01 Renal failure | 2 | 0.3% |
| 01.08 & 10.05 Tetanus | 1 | 0.2% |
| (blank) | 1 | 0.2% |
| 12.03 Accid fall | 1 | 0.2% |
| 06.02 Liver cirrhosis | 1 | 0.2% |
| 98 Other and unspecified NCD | 1 | 0.2% |
| 03.03 Diabetes mellitus | 1 | 0.2% |
| **Grand Total** | **630** | **100%** |

**S4B Table) Verbal autopsy assigned causes of death in Zambia among persons with HIV aged 12 years and above, 2020-2023**

| **Cause of Death** | **n** | **%** |
| --- | --- | --- |
| 01.03 HIV/AIDS related death | 5424 | 50.1% |
| Cardiac disease (code 04.01 & 04.99) | 1497 | 13.8% |
| 01.09 Pulmonary tuberculosis | 858 | 7.9% |
| 02.02 Digestive neoplasms | 445 | 4.1% |
| 01.02 Acute resp infect incl pneumonia | 323 | 3.0% |
| 04.02 Stroke | 315 | 2.9% |
| 03.03 Diabetes mellitus | 182 | 1.7% |
| 01.04 Diarrhoeal diseases | 175 | 1.6% |
| Indeterminate | 168 | 1.6% |
| 12.01 Road traffic accident | 160 | 1.5% |
| 06.02 Liver cirrhosis | 157 | 1.4% |
| 12.08 Intentional self-harm | 147 | 1.4% |
| 12.09 Assault | 143 | 1.3% |
| 02.03 Respiratory neoplasms | 132 | 1.2% |
| 02.05 & 02.06 Reproductive neoplasms MF | 92 | 0.8% |
| 08.01 Epilepsy | 73 | 0.7% |
| 02.99 Other and unspecified neoplasms | 61 | 0.6% |
| 01.99 Other and unspecified infect dis | 51 | 0.5% |
| 12.03 Accid fall | 49 | 0.5% |
| 07.01 Renal failure | 48 | 0.4% |
| 01.05 Malaria | 44 | 0.4% |
| 06.01 Acute abdomen | 43 | 0.4% |
| 01.07 Meningitis and encephalitis | 37 | 0.3% |
| 01.01 Sepsis (non-obstetric) | 33 | 0.3% |
| 09.04 Obstetric haemorrhage | 24 | 0.2% |
| 12.07 Accid poisoning & noxious subs | 21 | 0.2% |
| 12.99 Other and unspecified external CoD | 18 | 0.2% |
| 02.01 Oral neoplasms | 14 | 0.1% |
| 05.02 Asthma | 11 | 0.1% |
| 12.05 Accid expos to smoke fire & flame | 9 | 0.1% |
| 98 Other and unspecified NCD | 9 | 0.1% |
| 03.01 Severe anaemia | 9 | 0.1% |
| 12.04 Accid drowning and submersion | 9 | 0.1% |
| 02.04 Breast neoplasms | 9 | 0.1% |
| 09.02 Abortion-related death | 9 | 0.1% |
| 05.01 Chronic obstructive pulmonary dis | 6 | 0.1% |
| 09.03 Pregnancy-induced hypertension | 5 | 0.0% |
| 12.10 Exposure to force of nature | 5 | 0.0% |
| (blank) | 4 | 0.0% |
| 09.06 Pregnancy-related sepsis | 4 | 0.0% |
| 12.02 Other transport accident | 3 | 0.0% |
| 09.07 Anaemia of pregnancy | 2 | 0.0% |
| 09.08 Ruptured uterus | 2 | 0.0% |
| 03.02 Severe malnutrition | 2 | 0.0% |
| 09.99 Other and unspecified maternal CoD | 1 | 0.0% |
| 12.06 Contact with venomous plant/animal | 1 | 0.0% |
| 01.11 Haemorrhagic fever (non-dengue) | 1 | 0.0% |
| 01.12 Dengue fever | 1 | 0.0% |
| **Overall** | **10836** | **100%** |

**S5A Table) Verbal autopsy assigned causes of death stratified by place of death among all decedents in Zambia, 2020-2023**

|  | **Health Facility** | | **Home** | | **Other** | | **Overall** | |
| --- | --- | --- | --- | --- | --- | --- | --- | --- |
| **Cause of Death** | **n** | **%** | **n** | **%** | **n** | **%** | **n** | **%** |
| Cardiac disease (code 04.01 & 04.99) | 3315 | 20.5% | 11128 | 23.3% | 373 | 11.8% | 14816 | 22.1% |
| 01.03 HIV/AIDS related death | 1249 | 7.7% | 4480 | 9.4% | 60 | 1.9% | 5789 | 8.6% |
| 04.02 Stroke | 807 | 5.0% | 3914 | 8.2% | 63 | 2.0% | 4784 | 7.1% |
| 01.02 Acute resp infect incl pneumonia | 1534 | 9.5% | 2760 | 5.8% | 78 | 2.5% | 4372 | 6.5% |
| 01.04 Diarrhoeal diseases | 1476 | 9.1% | 2661 | 5.6% | 58 | 1.8% | 4195 | 6.3% |
| 02.02 Digestive neoplasms | 707 | 4.4% | 2513 | 5.3% | 38 | 1.2% | 3258 | 4.9% |
| 01.09 Pulmonary tuberculosis | 563 | 3.5% | 2295 | 4.8% | 50 | 1.6% | 2908 | 4.3% |
| Indeterminate | 565 | 3.5% | 1920 | 4.0% | 152 | 4.8% | 2637 | 3.9% |
| 03.03 Diabetes mellitus | 551 | 3.4% | 1912 | 4.0% | 32 | 1.0% | 2495 | 3.7% |
| 12.01 Road traffic accident | 352 | 2.2% | 882 | 1.8% | 852 | 27.0% | 2086 | 3.1% |
| 12.09 Assault | 185 | 1.1% | 1427 | 3.0% | 455 | 14.4% | 2067 | 3.1% |
| 01.99 Other and unspecified infect dis | 644 | 4.0% | 1017 | 2.1% | 18 | 0.6% | 1679 | 2.5% |
| 01.05 Malaria | 441 | 2.7% | 858 | 1.8% | 20 | 0.6% | 1319 | 2.0% |
| 12.08 Intentional self-harm | 222 | 1.4% | 806 | 1.7% | 119 | 3.8% | 1147 | 1.7% |
| 06.02 Liver cirrhosis | 281 | 1.7% | 840 | 1.8% | 12 | 0.4% | 1133 | 1.7% |
| 01.07 Meningitis and encephalitis | 357 | 2.2% | 747 | 1.6% | 16 | 0.5% | 1120 | 1.7% |
| 08.01 Epilepsy | 146 | 0.9% | 861 | 1.8% | 37 | 1.2% | 1044 | 1.6% |
| 02.03 Respiratory neoplasms | 193 | 1.2% | 809 | 1.7% | 22 | 0.7% | 1024 | 1.5% |
| 12.03 Accid fall | 127 | 0.8% | 510 | 1.1% | 87 | 2.8% | 724 | 1.1% |
| 02.99 Other and unspecified neoplasms | 86 | 0.5% | 604 | 1.3% | 10 | 0.3% | 700 | 1.0% |
| 10.01 Prematurity | 384 | 2.4% | 279 | 0.6% | 2 | 0.1% | 665 | 1.0% |
| 10.02 Birth asphyxia | 419 | 2.6% | 219 | 0.5% | 5 | 0.2% | 643 | 1.0% |
| 12.04 Accid drowning and submersion | 9 | 0.1% | 293 | 0.6% | 337 | 10.7% | 639 | 1.0% |
| 02.05 & 02.06 Reproductive neoplasms MF | 102 | 0.6% | 506 | 1.1% | 9 | 0.3% | 617 | 0.9% |
| 07.01 Renal failure | 133 | 0.8% | 402 | 0.8% | 5 | 0.2% | 540 | 0.8% |
| 06.01 Acute abdomen | 158 | 1.0% | 309 | 0.6% | 10 | 0.3% | 477 | 0.7% |
| 03.02 Severe malnutrition | 80 | 0.5% | 313 | 0.7% | 4 | 0.1% | 397 | 0.6% |
| 01.01 Sepsis (non-obstetric) | 100 | 0.6% | 281 | 0.6% | 6 | 0.2% | 387 | 0.6% |
| 12.99 Other and unspecified external CoD | 34 | 0.2% | 209 | 0.4% | 30 | 1.0% | 273 | 0.4% |
| 10.03 Neonatal pneumonia | 144 | 0.9% | 91 | 0.2% | 2 | 0.1% | 237 | 0.4% |
| 10.04 Neonatal sepsis | 84 | 0.5% | 152 | 0.3% | 1 | 0.0% | 237 | 0.4% |
| 12.07 Accid poisoning & noxious subs | 59 | 0.4% | 159 | 0.3% | 10 | 0.3% | 228 | 0.3% |
| 12.05 Accid expos to smoke fire & flame | 48 | 0.3% | 165 | 0.3% | 14 | 0.4% | 227 | 0.3% |
| 10.06 Congenital malformation | 99 | 0.6% | 124 | 0.3% | 2 | 0.1% | 225 | 0.3% |
| 09.04 Obstetric haemorrhage | 75 | 0.5% | 115 | 0.2% | 7 | 0.2% | 197 | 0.3% |
| 12.02 Other transport accident | 25 | 0.2% | 110 | 0.2% | 61 | 1.9% | 196 | 0.3% |
| 98 Other and unspecified NCD | 19 | 0.1% | 164 | 0.3% | 12 | 0.4% | 195 | 0.3% |
| 05.01 Chronic obstructive pulmonary dis | 23 | 0.1% | 120 | 0.3% | 0 | 0.0% | 143 | 0.2% |
| 12.10 Exposure to force of nature | 4 | 0.0% | 88 | 0.2% | 47 | 1.5% | 139 | 0.2% |
| 02.04 Breast neoplasms | 13 | 0.1% | 112 | 0.2% | 2 | 0.1% | 127 | 0.2% |
| 11.01 Fresh stillbirth | 88 | 0.5% | 25 | 0.1% | 2 | 0.1% | 115 | 0.2% |
| 02.01 Oral neoplasms | 7 | 0.0% | 84 | 0.2% | 1 | 0.0% | 92 | 0.1% |
| 11.02 Macerated stillbirth | 46 | 0.3% | 37 | 0.1% | 2 | 0.1% | 85 | 0.1% |
| 09.02 Abortion-related death | 39 | 0.2% | 45 | 0.1% | 1 | 0.0% | 85 | 0.1% |
| 05.02 Asthma | 24 | 0.1% | 57 | 0.1% | 4 | 0.1% | 85 | 0.1% |
| 01.08 & 10.05 Tetanus | 43 | 0.3% | 35 | 0.1% | 1 | 0.0% | 79 | 0.1% |
| (blank) | 13 | 0.1% | 50 | 0.1% | 5 | 0.2% | 68 | 0.1% |
| 03.01 Severe anaemia | 10 | 0.1% | 45 | 0.1% | 1 | 0.0% | 56 | 0.1% |
| 12.06 Contact with venomous plant/animal | 10 | 0.1% | 34 | 0.1% | 10 | 0.3% | 54 | 0.1% |
| 09.03 Pregnancy-induced hypertension | 16 | 0.1% | 32 | 0.1% | 1 | 0.0% | 49 | 0.1% |
| 01.10 Pertussis | 13 | 0.1% | 32 | 0.1% | 0 | 0.0% | 45 | 0.1% |
| 01.11 Haemorrhagic fever (non-dengue) | 17 | 0.1% | 21 | 0.0% | 3 | 0.1% | 41 | 0.1% |
| 01.12 Dengue fever | 19 | 0.1% | 21 | 0.0% | 0 | 0.0% | 40 | 0.1% |
| 09.06 Pregnancy-related sepsis | 6 | 0.0% | 26 | 0.1% | 0 | 0.0% | 32 | 0.0% |
| 04.03 Sickle cell with crisis | 5 | 0.0% | 12 | 0.0% | 0 | 0.0% | 17 | 0.0% |
| 09.01 Ectopic pregnancy | 6 | 0.0% | 8 | 0.0% | 0 | 0.0% | 14 | 0.0% |
| 09.99 Other and unspecified maternal CoD | 3 | 0.0% | 7 | 0.0% | 1 | 0.0% | 11 | 0.0% |
| 09.08 Ruptured uterus | 3 | 0.0% | 4 | 0.0% | 0 | 0.0% | 7 | 0.0% |
| 09.07 Anaemia of pregnancy | 3 | 0.0% | 4 | 0.0% | 0 | 0.0% | 7 | 0.0% |
| 01.06 Measles | 2 | 0.0% | 4 | 0.0% | 0 | 0.0% | 6 | 0.0% |
| 10.99 Other and unspecified neonatal CoD | 3 | 0.0% | 1 | 0.0% | 0 | 0.0% | 4 | 0.0% |
| 09.05 Obstructed labour | 0 | 0.0% | 1 | 0.0% | 0 | 0.0% | 1 | 0.0% |
| **Overall** | **16189** | **100%** | **47740** | **100%** | **3150** | **100%** | **67079** | **100%** |

**S5B Table) Verbal autopsy assigned causes of death in Zambia stratified by place of death among persons with HIV, 2020-2023**

|  | **Health Facility** | | **Home** | | **Other** | | **Overall** | |
| --- | --- | --- | --- | --- | --- | --- | --- | --- |
| **Cause of Death** | **n** | **%** | **n** | **%** | **n** | **%** | **n** | **%** |
| 01.03 HIV/AIDS related death | 1249 | 47.3% | 4480 | 52.4% | 60 | 20.9% | 5789 | 50.4% |
| Cardiac disease (code 04.01 & 04.99) | 404 | 15.3% | 1053 | 12.3% | 47 | 16.4% | 1504 | 13.1% |
| 01.09 Pulmonary tuberculosis | 181 | 6.9% | 667 | 7.8% | 13 | 4.5% | 861 | 7.5% |
| 02.02 Digestive neoplasms | 116 | 4.4% | 324 | 3.8% | 5 | 1.7% | 445 | 3.9% |
| 01.02 Acute resp infect incl pneumonia | 124 | 4.7% | 216 | 2.5% | 6 | 2.1% | 346 | 3.0% |
| 04.02 Stroke | 75 | 2.8% | 230 | 2.7% | 10 | 3.5% | 315 | 2.7% |
| 01.04 Diarrhoeal diseases | 85 | 3.2% | 173 | 2.0% | 7 | 2.4% | 265 | 2.3% |
| Indeterminate | 44 | 1.7% | 136 | 1.6% | 12 | 4.2% | 192 | 1.7% |
| 03.03 Diabetes mellitus | 42 | 1.6% | 139 | 1.6% | 2 | 0.7% | 183 | 1.6% |
| 12.01 Road traffic accident | 25 | 0.9% | 97 | 1.1% | 43 | 15.0% | 165 | 1.4% |
| 06.02 Liver cirrhosis | 40 | 1.5% | 117 | 1.4% | 1 | 0.3% | 158 | 1.4% |
| 12.08 Intentional self-harm | 25 | 0.9% | 107 | 1.3% | 15 | 5.2% | 147 | 1.3% |
| 12.09 Assault | 8 | 0.3% | 104 | 1.2% | 31 | 10.8% | 143 | 1.2% |
| 02.03 Respiratory neoplasms | 30 | 1.1% | 96 | 1.1% | 6 | 2.1% | 132 | 1.2% |
| 02.05 & 02.06 Reproductive neoplasms MF | 11 | 0.4% | 81 | 0.9% | 0 | 0.0% | 92 | 0.8% |
| 01.99 Other and unspecified infect dis | 29 | 1.1% | 51 | 0.6% | 3 | 1.0% | 83 | 0.7% |
| 08.01 Epilepsy | 16 | 0.6% | 61 | 0.7% | 3 | 1.0% | 80 | 0.7% |
| 02.99 Other and unspecified neoplasms | 10 | 0.4% | 51 | 0.6% | 0 | 0.0% | 61 | 0.5% |
| 01.07 Meningitis and encephalitis | 19 | 0.7% | 41 | 0.5% | 0 | 0.0% | 60 | 0.5% |
| 01.05 Malaria | 19 | 0.7% | 36 | 0.4% | 1 | 0.3% | 56 | 0.5% |
| 12.03 Accid fall | 5 | 0.2% | 35 | 0.4% | 10 | 3.5% | 50 | 0.4% |
| 07.01 Renal failure | 11 | 0.4% | 37 | 0.4% | 2 | 0.7% | 50 | 0.4% |
| 06.01 Acute abdomen | 14 | 0.5% | 28 | 0.3% | 1 | 0.3% | 43 | 0.4% |
| 01.01 Sepsis (non-obstetric) | 6 | 0.2% | 29 | 0.3% | 0 | 0.0% | 35 | 0.3% |
| 09.04 Obstetric haemorrhage | 6 | 0.2% | 18 | 0.2% | 0 | 0.0% | 24 | 0.2% |
| 03.02 Severe malnutrition | 5 | 0.2% | 18 | 0.2% | 0 | 0.0% | 23 | 0.2% |
| 12.07 Accid poisoning & noxious subs | 5 | 0.2% | 15 | 0.2% | 1 | 0.3% | 21 | 0.2% |
| 12.99 Other and unspecified external CoD | 3 | 0.1% | 15 | 0.2% | 0 | 0.0% | 18 | 0.2% |
| 02.01 Oral neoplasms | 3 | 0.1% | 11 | 0.1% | 0 | 0.0% | 14 | 0.1% |
| 12.04 Accid drowning and submersion | 0 | 0.0% | 9 | 0.1% | 4 | 1.4% | 13 | 0.1% |
| 05.02 Asthma | 4 | 0.2% | 7 | 0.1% | 0 | 0.0% | 11 | 0.1% |
| 12.05 Accid expos to smoke fire & flame | 2 | 0.1% | 9 | 0.1% | 0 | 0.0% | 11 | 0.1% |
| 98 Other and unspecified NCD | 0 | 0.0% | 10 | 0.1% | 0 | 0.0% | 10 | 0.1% |
| 09.02 Abortion-related death | 3 | 0.1% | 6 | 0.1% | 0 | 0.0% | 9 | 0.1% |
| 03.01 Severe anaemia | 0 | 0.0% | 9 | 0.1% | 0 | 0.0% | 9 | 0.1% |
| 02.04 Breast neoplasms | 0 | 0.0% | 9 | 0.1% | 0 | 0.0% | 9 | 0.1% |
| 05.01 Chronic obstructive pulmonary dis | 5 | 0.2% | 1 | 0.0% | 0 | 0.0% | 6 | 0.1% |
| 12.10 Exposure to force of nature | 0 | 0.0% | 4 | 0.0% | 1 | 0.3% | 5 | 0.0% |
| 09.03 Pregnancy-induced hypertension | 1 | 0.0% | 4 | 0.0% | 0 | 0.0% | 5 | 0.0% |
| (blank) | 2 | 0.1% | 2 | 0.0% | 1 | 0.3% | 5 | 0.0% |
| 09.06 Pregnancy-related sepsis | 1 | 0.0% | 3 | 0.0% | 0 | 0.0% | 4 | 0.0% |
| 10.06 Congenital malformation | 1 | 0.0% | 3 | 0.0% | 0 | 0.0% | 4 | 0.0% |
| 12.02 Other transport accident | 0 | 0.0% | 1 | 0.0% | 2 | 0.7% | 3 | 0.0% |
| 01.12 Dengue fever | 1 | 0.0% | 2 | 0.0% | 0 | 0.0% | 3 | 0.0% |
| 09.08 Ruptured uterus | 1 | 0.0% | 1 | 0.0% | 0 | 0.0% | 2 | 0.0% |
| 10.01 Prematurity | 2 | 0.1% | 0 | 0.0% | 0 | 0.0% | 2 | 0.0% |
| 01.10 Pertussis | 2 | 0.1% | 0 | 0.0% | 0 | 0.0% | 2 | 0.0% |
| 09.07 Anaemia of pregnancy | 0 | 0.0% | 2 | 0.0% | 0 | 0.0% | 2 | 0.0% |
| 09.99 Other and unspecified maternal CoD | 1 | 0.0% | 0 | 0.0% | 0 | 0.0% | 1 | 0.0% |
| 01.08 & 10.05 Tetanus | 1 | 0.0% | 0 | 0.0% | 0 | 0.0% | 1 | 0.0% |
| 12.06 Contact with venomous plant/animal | 0 | 0.0% | 1 | 0.0% | 0 | 0.0% | 1 | 0.0% |
| 10.03 Neonatal pneumonia | 0 | 0.0% | 1 | 0.0% | 0 | 0.0% | 1 | 0.0% |
| 01.11 Haemorrhagic fever (non-dengue) | 1 | 0.0% | 0 | 0.0% | 0 | 0.0% | 1 | 0.0% |
| **Overall** | **2638** | **100%** | **8550** | **100%** | **287** | **100%** | **11475** | **100%** |

**S5C Table) Verbal autopsy assigned causes of death in Zambia stratified by place of death among persons without HIV, 2020-2023**

|  | **Health Facility** | | **Home** | | **Other** | | **Overall** | |
| --- | --- | --- | --- | --- | --- | --- | --- | --- |
| **Cause of Death** | **n** | **%** | **n** | **%** | **n** | **%** | **n** | **%** |
| Cardiac disease (code 04.01 & 04.99) | 2791 | 23.6% | 9726 | 26.3% | 303 | 11.4% | 12820 | 24.9% |
| 04.02 Stroke | 709 | 6.0% | 3609 | 9.8% | 50 | 1.9% | 4368 | 8.5% |
| 01.02 Acute resp infect incl pneumonia | 1363 | 11.5% | 2458 | 6.6% | 65 | 2.4% | 3886 | 7.6% |
| 01.04 Diarrhoeal diseases | 1368 | 11.6% | 2421 | 6.5% | 50 | 1.9% | 3839 | 7.5% |
| 02.02 Digestive neoplasms | 568 | 4.8% | 2098 | 5.7% | 32 | 1.2% | 2698 | 5.2% |
| Indeterminate | 462 | 3.9% | 1668 | 4.5% | 130 | 4.9% | 2260 | 4.4% |
| 03.03 Diabetes mellitus | 499 | 4.2% | 1731 | 4.7% | 28 | 1.1% | 2258 | 4.4% |
| 01.09 Pulmonary tuberculosis | 344 | 2.9% | 1516 | 4.1% | 32 | 1.2% | 1892 | 3.7% |
| 12.01 Road traffic accident | 314 | 2.7% | 746 | 2.0% | 758 | 28.5% | 1818 | 3.5% |
| 12.09 Assault | 167 | 1.4% | 1260 | 3.4% | 387 | 14.5% | 1814 | 3.5% |
| 01.99 Other and unspecified infect dis | 604 | 5.1% | 933 | 2.5% | 15 | 0.6% | 1552 | 3.0% |
| 01.05 Malaria | 412 | 3.5% | 792 | 2.1% | 19 | 0.7% | 1223 | 2.4% |
| 01.07 Meningitis and encephalitis | 323 | 2.7% | 688 | 1.9% | 16 | 0.6% | 1027 | 2.0% |
| 12.08 Intentional self-harm | 186 | 1.6% | 661 | 1.8% | 95 | 3.6% | 942 | 1.8% |
| 08.01 Epilepsy | 120 | 1.0% | 780 | 2.1% | 32 | 1.2% | 932 | 1.8% |
| 06.02 Liver cirrhosis | 231 | 2.0% | 687 | 1.9% | 11 | 0.4% | 929 | 1.8% |
| 02.03 Respiratory neoplasms | 152 | 1.3% | 684 | 1.9% | 13 | 0.5% | 849 | 1.7% |
| 12.03 Accid fall | 117 | 1.0% | 467 | 1.3% | 72 | 2.7% | 656 | 1.3% |
| 02.99 Other and unspecified neoplasms | 73 | 0.6% | 537 | 1.5% | 7 | 0.3% | 617 | 1.2% |
| 12.04 Accid drowning and submersion | 9 | 0.1% | 281 | 0.8% | 323 | 12.1% | 613 | 1.2% |
| 02.05 & 02.06 Reproductive neoplasms MF | 84 | 0.7% | 411 | 1.1% | 8 | 0.3% | 503 | 1.0% |
| 07.01 Renal failure | 115 | 1.0% | 355 | 1.0% | 3 | 0.1% | 473 | 0.9% |
| 06.01 Acute abdomen | 132 | 1.1% | 266 | 0.7% | 7 | 0.3% | 405 | 0.8% |
| 03.02 Severe malnutrition | 75 | 0.6% | 289 | 0.8% | 4 | 0.2% | 368 | 0.7% |
| 01.01 Sepsis (non-obstetric) | 92 | 0.8% | 251 | 0.7% | 6 | 0.2% | 349 | 0.7% |
| 12.99 Other and unspecified external CoD | 31 | 0.3% | 183 | 0.5% | 29 | 1.1% | 243 | 0.5% |
| 12.05 Accid expos to smoke fire & flame | 44 | 0.4% | 151 | 0.4% | 14 | 0.5% | 209 | 0.4% |
| 12.07 Accid poisoning & noxious subs | 51 | 0.4% | 137 | 0.4% | 8 | 0.3% | 196 | 0.4% |
| 12.02 Other transport accident | 22 | 0.2% | 102 | 0.3% | 59 | 2.2% | 183 | 0.4% |
| 98 Other and unspecified NCD | 18 | 0.2% | 151 | 0.4% | 12 | 0.5% | 181 | 0.4% |
| 09.04 Obstetric haemorrhage | 66 | 0.6% | 90 | 0.2% | 6 | 0.2% | 162 | 0.3% |
| 10.06 Congenital malformation | 35 | 0.3% | 99 | 0.3% | 0 | 0.0% | 134 | 0.3% |
| 05.01 Chronic obstructive pulmonary dis | 18 | 0.2% | 115 | 0.3% | 0 | 0.0% | 133 | 0.3% |
| 12.10 Exposure to force of nature | 4 | 0.0% | 84 | 0.2% | 40 | 1.5% | 128 | 0.2% |
| 02.04 Breast neoplasms | 13 | 0.1% | 100 | 0.3% | 2 | 0.1% | 115 | 0.2% |
| 01.08 & 10.05 Tetanus | 42 | 0.4% | 34 | 0.1% | 1 | 0.0% | 77 | 0.1% |
| 02.01 Oral neoplasms | 4 | 0.0% | 70 | 0.2% | 1 | 0.0% | 75 | 0.1% |
| 09.02 Abortion-related death | 35 | 0.3% | 35 | 0.1% | 1 | 0.0% | 71 | 0.1% |
| 05.02 Asthma | 19 | 0.2% | 48 | 0.1% | 4 | 0.2% | 71 | 0.1% |
| 12.06 Contact with venomous plant/animal | 10 | 0.1% | 33 | 0.1% | 10 | 0.4% | 53 | 0.1% |
| 03.01 Severe anaemia | 9 | 0.1% | 35 | 0.1% | 1 | 0.0% | 45 | 0.1% |
| 01.10 Pertussis | 11 | 0.1% | 32 | 0.1% | 0 | 0.0% | 43 | 0.1% |
| 09.03 Pregnancy-induced hypertension | 14 | 0.1% | 27 | 0.1% | 1 | 0.0% | 42 | 0.1% |
| 01.11 Haemorrhagic fever (non-dengue) | 14 | 0.1% | 21 | 0.1% | 3 | 0.1% | 38 | 0.1% |
| 01.12 Dengue fever | 18 | 0.2% | 19 | 0.1% | 0 | 0.0% | 37 | 0.1% |
| (blank) | 4 | 0.0% | 28 | 0.1% | 3 | 0.1% | 35 | 0.1% |
| 09.06 Pregnancy-related sepsis | 3 | 0.0% | 22 | 0.1% | 0 | 0.0% | 25 | 0.0% |
| 04.03 Sickle cell with crisis | 5 | 0.0% | 12 | 0.0% | 0 | 0.0% | 17 | 0.0% |
| 09.01 Ectopic pregnancy | 6 | 0.1% | 8 | 0.0% | 0 | 0.0% | 14 | 0.0% |
| 09.99 Other and unspecified maternal CoD | 2 | 0.0% | 7 | 0.0% | 1 | 0.0% | 10 | 0.0% |
| 01.06 Measles | 2 | 0.0% | 4 | 0.0% | 0 | 0.0% | 6 | 0.0% |
| 09.08 Ruptured uterus | 2 | 0.0% | 3 | 0.0% | 0 | 0.0% | 5 | 0.0% |
| 09.07 Anaemia of pregnancy | 3 | 0.0% | 2 | 0.0% | 0 | 0.0% | 5 | 0.0% |
| 09.05 Obstructed labour | 0 | 0.0% | 1 | 0.0% | 0 | 0.0% | 1 | 0.0% |
| **Overall** | **11815** | **100%** | **36968** | **100%** | **2662** | **100%** | **51445** | **100%** |
